# Brain region-specific and systemic transcriptomic alterations in a human alpha-synuclein overexpressing rat model

**DOI:** 10.18632/aging.206331

**Published:** 2025-10-20

**Authors:** Vivien Hoof, Nicolas Casadei, Olaf Riess, Julia Schulze-Hentrich, Thomas Hentrich

**Affiliations:** 1 Department of Genetics/Epigenetics, Saarland University, Saarbrücken, Germany; 2 Institute of Medical Genetics and Applied Genomics, University of Tübingen, Tübingen, Germany; 3 NGS Competence Center Tübingen, Tübingen, Germany

**Keywords:** alpha-synuclein, transgenic rat model, different brain regions, aging, transcriptome analysis

## Abstract

Synucleinopathies are age-dependent neurodegenerative diseases characterized by alpha-synuclein accumulation with distinct vulnerabilities across brain regions. Understanding early disease stages is essential to uncover initial molecular changes that might enable earlier diagnosis and causal therapy. In this study, we profiled longitudinal and brain region-resolved gene expression changes in a rat model of synucleinopathies overexpressing human *SNCA*. Transcriptomic analyses were performed on gene and transcript level of striatal, frontocortical, and cerebellar tissue in 5- and 12-month-old transgenic (BAC SNCA) and wild type rats revealing that *SNCA* overexpression leads to age-dependent transcriptomic changes that largely occur region-specific. In frontal cortex, dysregulation of myelination-associated genes agreed with Parkinson patient data as shown before. In addition, BAC SNCA rats displayed more gene expression changes at younger age, with a common and characteristic alteration pattern across all three examined brain regions. We also identified a cross-regional set of differential genes that were affected by *SNCA* overload. This set was also partially reflected in the gut transcriptome of the same rat model, suggesting a systemic impact of *SNCA* overload. Taken together, our findings highlight both brain region-specific vulnerabilities and global molecular perturbations associated with alpha-synuclein biology and provide insights into early transcriptomic changes in synucleinopathies.

## INTRODUCTION

Synucleinopathies, including Parkinson’s disease (PD), dementia with Lewy bodies (DLB), and multiple system atrophy (MSA), are neurodegenerative disorders characterized by increased accumulation of misfolded alpha-synuclein (aSyn) in Lewy bodies, Lewy neurites or glial inclusions [1, 2]. Alpha-synuclein, encoded by the *SNCA* gene, is a neural protein, predominantly located at the presynapse [3]. Its cellular function has not been fully elucidated, but it is implicated in regulating neurotransmitter release and synaptic function. Accumulations of aSyn can impair presynaptic vesicle fusion and release of neurotransmitters [4]. Genetic studies point to the pivotal pathogenic role of aSyn in synucleinopathies since point mutations and genomic multiplications of *SNCA* are associated with familial cases of PD [5–10]. Even in seemingly sporadic cases—which account for the majority—aSyn aggregations are the pathological hallmark [1].

The risk of developing synucleinopathies likely results from a complex interplay of genetic predisposition, aging, and environmental factors, with age being the highest risk factor, as reflected by increasing prevalence in elderly [11, 12]. Clinical diagnosis of synucleinopathies is based on motor symptoms that typically surface late during disease progression when neurodegeneration has already advanced. However, prodromal symptoms, such as smell loss, constipation, and REM sleep behaviour disorder often surface years prior to first motor impairments but lack diagnostic specificity [13, 14]. The prodromal stage of synucleinopathies suggests an early unfolding of pathology, based on underlying molecular alterations. Thus, investigating early time points is critical for understanding molecular dysregulations which could enable much-needed earlier diagnosis [15].

Since human brain samples typically only reflect the terminal point of the disease, animal models remain an important tool to investigate pathomechanisms in early stages of synucleinopathies. Previous studies have demonstrated that transcriptome alterations occur in both patients of synucleinopathies as well as animal models [16–18]. In this study, a transgenic (TG) rat model overexpressing full-length human wild type *SNCA* on a BAC/PAC fusion construct was used, as slow disease progression and underlying pathological changes in this model resemble aspects of human pathology [19, 20]. Progressive accumulation of aSyn in TG rats leads to age-dependent neural degeneration, with early phenotypic changes in novelty-seeking, avoidance, smell, and late-onset locomotor impairments [19]. These age-dependent phenotypic changes are likely due to molecular perturbations which may include transcriptomic dysregulations caused by *SNCA* overexpression. Therefore, this study investigated gene expression changes in BAC SNCA rats at a pre-symptomatic and symptomatic stage to gain further insights into age-dependent transcriptomic alterations in synucleinopathies.

In addition, it is known that brain regions not only differ in susceptibility to aSyn pathology [21], driven by differences in cell type composition and neuronal connectivity [22], but also ages largely region-specifically [23]. Hence, region-resolved and longitudinal analyses of expression changes are required to better understand the pathogenesis. That is why we interrogated striatal, frontocortical, and cerebellar tissue of 5- and 12-month-old TG rats as well as WT controls by RNA-sequencing. Differential analysis on gene and transcript level revealed age-dependent transcriptomic changes in *SNCA* overexpressing rats that largely occur brain region-specifically and partially agree with human data. Furthermore, a core set of genes shared similar perturbation patterns across brain regions and extended in parts into gut tissue.

## RESULTS

### Overexpression of human *SNCA* transcripts differs between brain regions in rat

**Figure 1:**
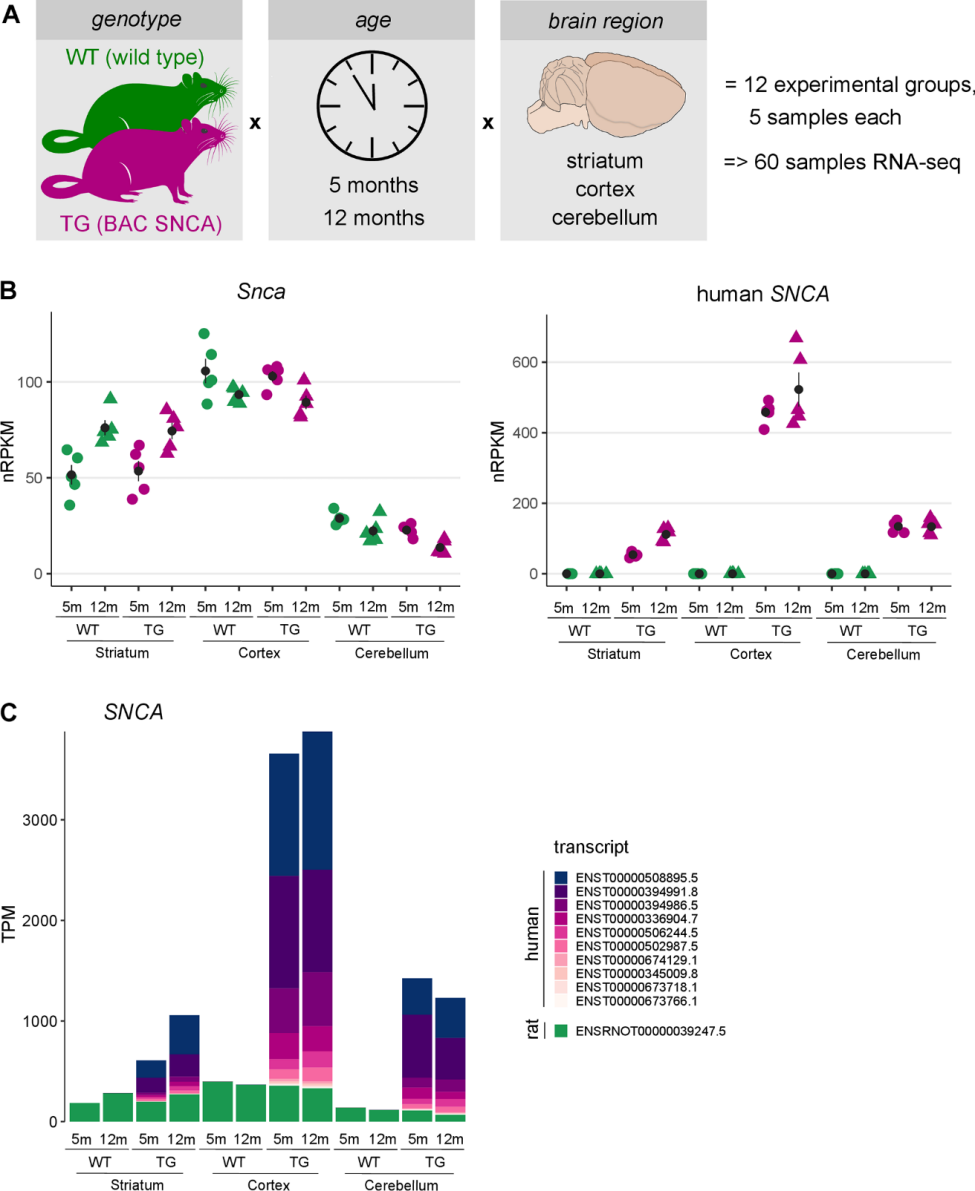
**Brain region-specific overexpression of endogenous and human *SNCA* in rats.** (**A**) Schematic illustration showing the experimental design of 5- and 12-months-old wildtype (WT) and transgenic (TG) BAC SNCA rats. RNA was isolated from striatum, cortex and cerebellum for 5 animals per group and used for RNA-seq to identify differential expression. (**B**) Expression level of endogenous rat *Snca* (left) and human *SNCA* (right) as individual nRPKM data points per rat across experimental groups with mean and standard error of the mean. (**C**) Composition and expression level of endogenous and human *SNCA* transcript isoforms across experimental groups.

To better understand effects of *SNCA* overexpression in brain regions, the striatal, frontocortical, and cerebellar transcriptomes of transgenic rats (TG) overexpressing full-length human *SNCA,* and wild type (WT) controls were interrogated using RNA-sequencing. By including 5- and 12-month-old rats of both genotypes a particular emphasize was put on revealing longitudinal transcriptomic changes (Figure 1A). After preprocessing the raw data using well-established, community approved pipelines [24, 25], high quality of the data was ensured by stringent quality controls. Principal component analysis showed that gene expression differed strongly between brain regions (Supplementary Figure 1A), age and genotype (Supplementary Figures 1B, 3A, 5A, 7A). In line, cell type-specific single-cell reference data [26] indicate cell type composition differences between brain regions, most prominent with respect to microglia, pyramidal neurons, and oligodendrocytes. Within each brain region, experimental groups reflect great homogeneity suggesting that transcriptomic differences are unlikely to result from compositional shifts (Supplementary Figure 2).

Since aSyn protein load differs between brain regions in this model [19], expression of endogenous and human *SNCA* were analysed. Endogenous *Snca* showed the highest expression in cortex and lowest in cerebellum and remained largely stable with respect to age and genotype. In striatum, however, endogenous *Snca* showed a significant age-dependent expression increase in both WT and TG animals (Figure 1B). On top of endogenous *Snca* levels, a region-specific expression of human *SNCA* was detected with highest expression in cortical samples of TG rats. Like the endogenous *Snca*, expression levels of human *SNCA* were not affected by age in cortex and cerebellum, whereas a significant age-dependent increase was observed in striatum (Figure 1B). Furthermore, expression of *SNCA* transcripts was analysed, revealing an intriguing addition of human transcripts on top of endogenous *Snca* in TG rats. Although no compositional differences of *SNCA* isoforms were observed, relative isoform expression differed significantly across brain regions, dominated by ENST00000508895.5 and ENST00000394991.8 transcripts (Figure 1C).

These results indicate that human *SNCA* is expressed in an age- and region-specific manner that follows the expression of endogenous *Snca* on both gene and transcript level, lending the model to learn more about aSyn biology under normal and disease-like conditions.

### Striatal transcriptomic changes under *SNCA* overexpression largely occur already in young animals

**Figure 2:**
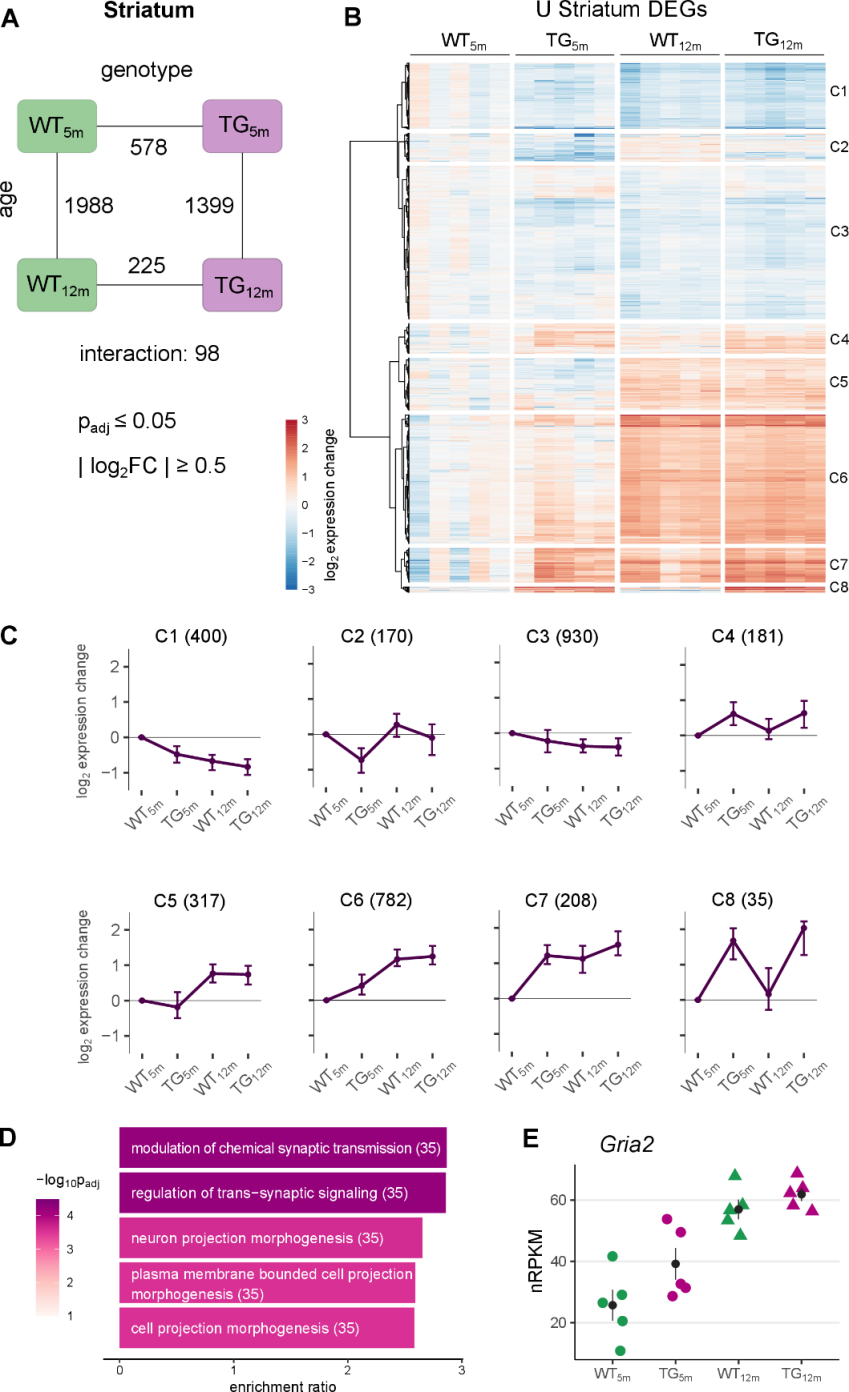
**Striatal gene expression changes under *SNCA* overload largely occur in young TG rats.** (**A**) Number of differentially expressed genes (DEGs) between experimental groups in the striatum, along the genotype (WT and TG) and age axes (5 and 12 months) and their interaction with the indicated significance cut-offs. (**B**) Heatmap of hierarchically clustered striatal expression changes relative to WT_5m_ of 2776 DEGs (union of striatal DEGs shown in Figure 2A) across experimental groups. (**C**) Average gene expression changes and standard deviation of all striatal DEGs plotted as centroids clustered in eight groups. Numbers of DEGs shown in brackets. (**D**) Five most significant enriched biological processes for the DEGs in cluster 6 with indicated adjusted p-value, enrichment ratio and DEG count in brackets. (**E**) Expression level for *Gria2* as individual nRPKM data points across experimental groups with mean and standard error of the mean.

Since the striatum is notably affected by aSyn pathology, particularly in PD [22, 27], the striatal transcriptome was analysed first. Globally, samples partitioned according to experimental conditions (Supplementary Figure 3A), and differential expression was determined with respect to age, genotype, and their interaction. Surprisingly, more differential expression was detected in younger than older TG rats, with 578 differentially expressed genes (DEGs) (369 up- and 209 downregulated) in 5-month-old and 225 DEGs (127 up- and 98 downregulated) in 12-month-old rats, of which 125 DEGs overlapped (Figure 2A and Supplementary Figure 3B). The counter-intuitive numbers of differential genes for an age-related disease also extended to transcript level (Supplementary Figure 3E) and cannot be attributed to higher inter-individual variability of the data in older animals (Supplementary Figure 3F). Also, differential splice event counts were higher in young TG rats, dominated by skipped exons, and occurring in genes associated with cytoplasm and synapse. Despite splicing events were largely distinct, there were few reoccurring genes with respect to genotype and age, respectively (Supplementary Figure 18A–18D).

The differential genes in 5-month-old TG rats were most significantly enriched for monovalent inorganic cation transport and sodium ion transport (Supplementary Figure 3C), whereas the DEGs in 12-month-old TG rats were neither significantly enriched for Gene Ontology terms, KEGG pathways, nor related annotations (Supplementary Figure 3D).

Trying to relate the differential entities with other models, even the striatal transcriptome of 6-months old mice overexpressing the same BAC SNCA construct showed limited overlap in differential genes (Supplementary Figure 4). Similar for RNA-seq data from putamen of PD patients [28], while indeed 83 orthologues were shared between TG rats and patients, similarity in directionality and magnitude was restricted to few genes only, including *LILRA5* and *TNNI3* as the most significantly up- and downregulated genes (Supplementary Figure 3G, 3H).

With respect to age, 1988 DEGs (971 up- and 1017 downregulated) were identified in WT and 1399 DEGs (482 up- and 917 downregulated) in TG rats (Figure 2A), suggesting that aging under aSyn overload differs between genotypes, which is statistically modeled by the interaction term that captured 98 DEGs (35 up- 63 downregulated) showing an interaction between age and genotype.

Borrowing reference data for striatal aging in mice [23], the expression patterns of these signature genes in the data formed four dominant clusters (Supplementary Figure 5). Cluster A2 and A3 contained genes that were either up- or downregulated specifically in TG rats, indicating an age-independent effect of the overexpressed transgene. In addition, the largest cluster (A4) contained genes showing an age-dependent upregulation largely irrespective of genotype. Furthermore, genes showing the strongest age-dependent upregulation, clustered separately in A1 because young TG rats reflected premature upregulation of those genes.

Broadening the view on these distinct expression patterns, DEGs from all striatal contrasts were merged, visualized in a heatmap and hierarchically clustered, revealing eight distinct groups (Figure 2B), summarized as centroids (Figure 2C). These clusters contained genes of analogous expression patterns described before (C4, C5 and C8). Furthermore, the largest clusters contained genes showing a premature up- (C6) and downregulation (C1 and C3) meaning they are differential in 5-month-old rats only (Supplementary Figure 6). Thus, premature alterations largely explain the high DEG count in young animals. Functionally, the 782 DEGs identified in cluster C6 were enriched for biological processes linked to synaptic functions including >modulation of chemical synaptic transmission and regulation of trans-synaptic signalling (Figure 2D), including *Gria2* which encodes for a glutamate ionotropic receptor with an important role in excitatory synaptic transmission [29] (Figure 2E). In addition, premature upregulated genes were enriched for targets of the transcription factor Pax6, which showed increased activity and is known to regulate neuronal development and neuronal plasticity [30] (Supplementary Figure 7).

Taken together, these results indicate greater perturbation of the striatal transcriptome in young TG animals. At 12 months, expression differences of these genes have largely converged again such that DEGs are fewer.

### Modest impact of *SNCA* overexpression on cerebellar transcriptome

**Figure 3:**
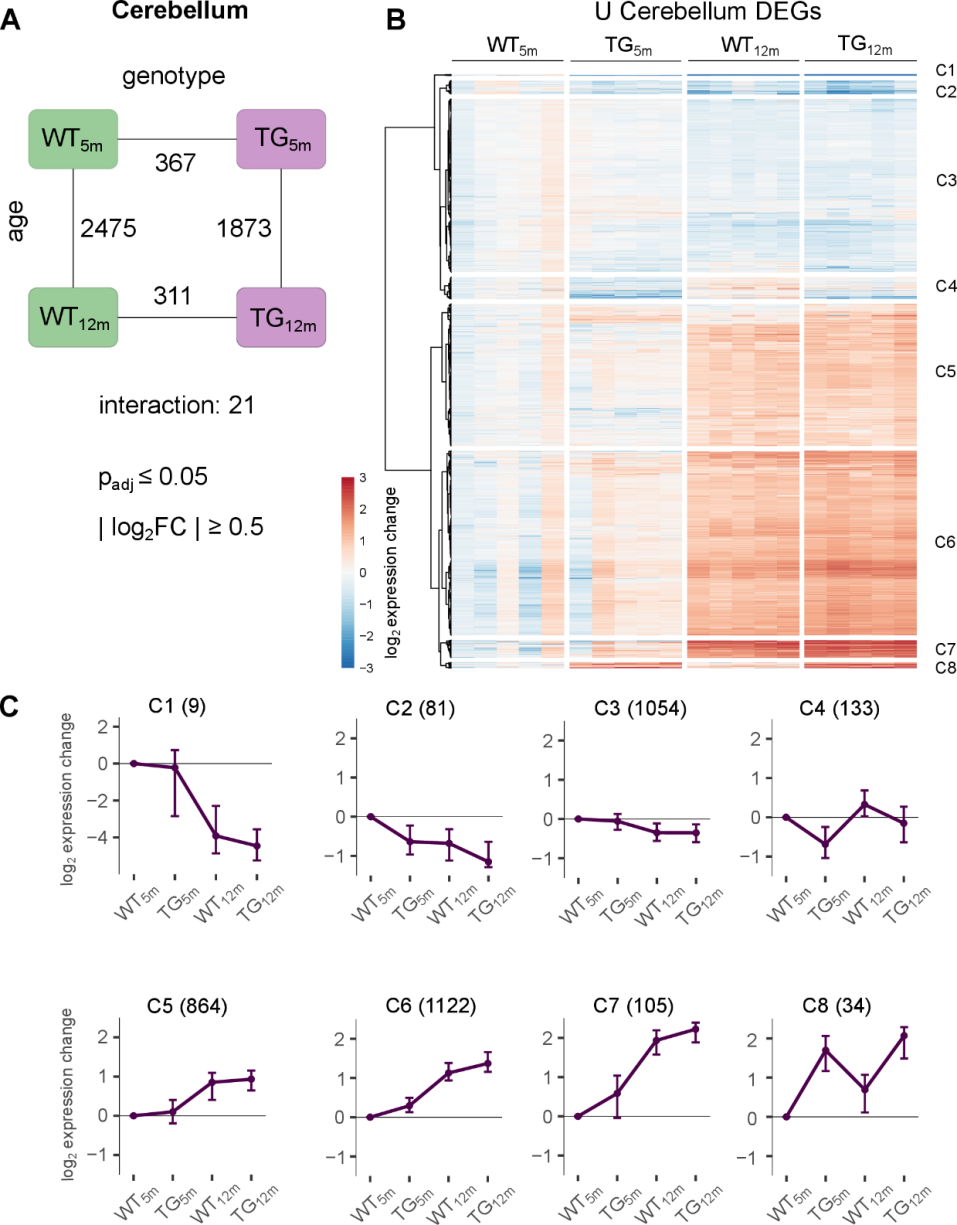
**Modest impact of *SNCA* overexpression on cerebellar transcriptome.** (**A**) Number of DEGs between experimental groups in the cerebellum, along the genotype (WT and TG) and age axes (5 and 12 months) and their interaction with the indicated significance cut-offs. (**B**) Heatmap of hierarchically clustered cerebellar expression changes of 3402 DEGs relative to WT_5m_ (union of cerebellar DEGs shown in Figure 3A) across experimental groups. (**C**) Average gene expression changes and standard deviation of all cerebellar DEGs plotted as centroids clustered in eight groups. Numbers of DEGs are shown in brackets.

To investigate whether transcriptomic changes occurring largely in young TG rats is specific to the striatum, analyses were extended to cortical and cerebellar samples. While the PCA of the cerebellar samples also indicates a genotype effect similar to striatum, samples separate predominantly by age (Supplementary Figure 8A). In line, differential analysis revealed 2475 DEGs (1719 up- and 756 downregulated) in WT and 1873 DEGs (1236 up- and 637 downregulated) in TG animals with respect to age, of which 1405 overlapped (Figure 3A and Supplementary Figure 8B). With respect to genotype, a similar DEG count of 367 (203 up- and 164 downregulated) and 311 (166 up- and 145 downregulated) were detected in 5- and 12-month-old TG rats, respectively (Figure 3A), with *Sncg*, *Gstt4*, and *Col27a1* being the most significantly upregulated and *P2rx4*, *Rtn4ip1*, and *Evc* the most significantly downregulated DEGs in TG animals. Of note, the DEGs of TG rats could neither be characterized by significant enrichments for Gene Ontology terms, KEGG pathways, and related annotation data. Differential splice events were similar to striatal observations, in which skipped exons dominated all contrasted conditions, occurred in genes annotated for cytoplasm, and were otherwise largely disjunct with the exception of a few reoccurring genes with respect to age and genotype (Supplementary Figure 18E–18H).

Due to the lack of cerebellar PD patient data, relating differential genes to the human condition remains for future studies. With respect to other models, genotype-driven DEGs partially overlapped with genes identified in cerebellum of PLP-alpha-synuclein transgenic mice, with *Sncg* being most significantly upregulated and *Chst5* being the most significantly downregulated in both models at 5 and 2 months of age and *Sorc2* and *Casq1* being most significantly up- respectively downregulated in both models and 12 months of age (Supplementary Table 1).

The similar count of differential genes in the cerebellum of BAC SNCA rats at 5- and 12-months of age also extends to transcript level (Supplementary Figure 8C). Together, the similarity of DEGs with respect to age in both genotypes and the stable impact of the transgene agree with only 21 genes (7 up- and 14 downregulated) that show significant interaction between age and genotype, suggesting only modest deviation on how WT and TG rats aged (Figure 3A). Yet again, signature genes of cerebellar aging showed similar patterns that were observed for striatal reference genes (Supplementary Figure 9), most importantly a cluster of genes showing premature upregulation in TG rats (A1 in Supplementary Figure 9B).

Investigating this further, the union of all cerebellar DEGs were visualized across experimental groups (Figure 3B), revealing expression patterns that clustered into eight distinct groups (Figure 3B, 3C and Supplementary Figure 10). Similar to striatum, two clusters (C5 and C3) comprise DEGs that show age-dependent up- and downregulation, respectively, largely independent of genotype. In addition, there are two cluster (C4 and C8) which show stable genotype driven perturbations (C4 and C8). Lastly, the largest number of genes were partitioned into clusters C6 and C7, showing similar activation with age in both genotypes, but premature upregulation in young TG animals. However, despite of clear and homogenous expression profiles, these DEGs were not significant enriched for any biological processes and pathways.

It should be noted that despite stronger overexpression of the transgene in cerebellum and the large DEG count in cerebellar contrasts their effect sizes tended to be weaker compared to striatal DEGs (Supplementary Figure 8D), suggesting that *SNCA* overexpression has a weaker impact on the aging cerebellar transcriptome.

### Myelination-linked genes were differential in cortex of BAC SNCA rats and PD patients

In a next step, cortical samples of both time points and genotypes were examined (Supplementary Figure 11A). Whereas in 5-month-old TG rats 610 DEGs (362 up- and 248 downregulated) were identified, 255 genes (169 up- and 86 downregulated) were differential in 12-month-old TG animals. Having more perturbations in young TG animals—similar to results in striatum (Figure 2)—also extended to transcript level and splice event counts (Supplementary Figures 11C, 18I) and was, again, not due to increased data variability in older animals (Supplementary Figure 11D). Also similar to striatal observations, differential splice events occurred mostly disjunct across contrasts, and were enriched for cytoplasm-linked genes (Supplementary Figure 18J, 18K).

With respect to age, 1385 DEGs (1016 up- and 369 downregulated) were detected in WT animals, whereas only 526 genes (436 up- and 90 downregulated) were differential in TG rats, with 315 DEGs overlapping (Figure 3A and Supplementary Figure 11B). As suggested by these numbers cortical aging under *SNCA* overexpression differed significantly from WT animals, most prominently captured in 197 DEGs (79 up- and 118 downregulated) showing a significant interaction between age and genotype, the highest number of all three examined brain regions (Figure 3A).

**Figure 4:**
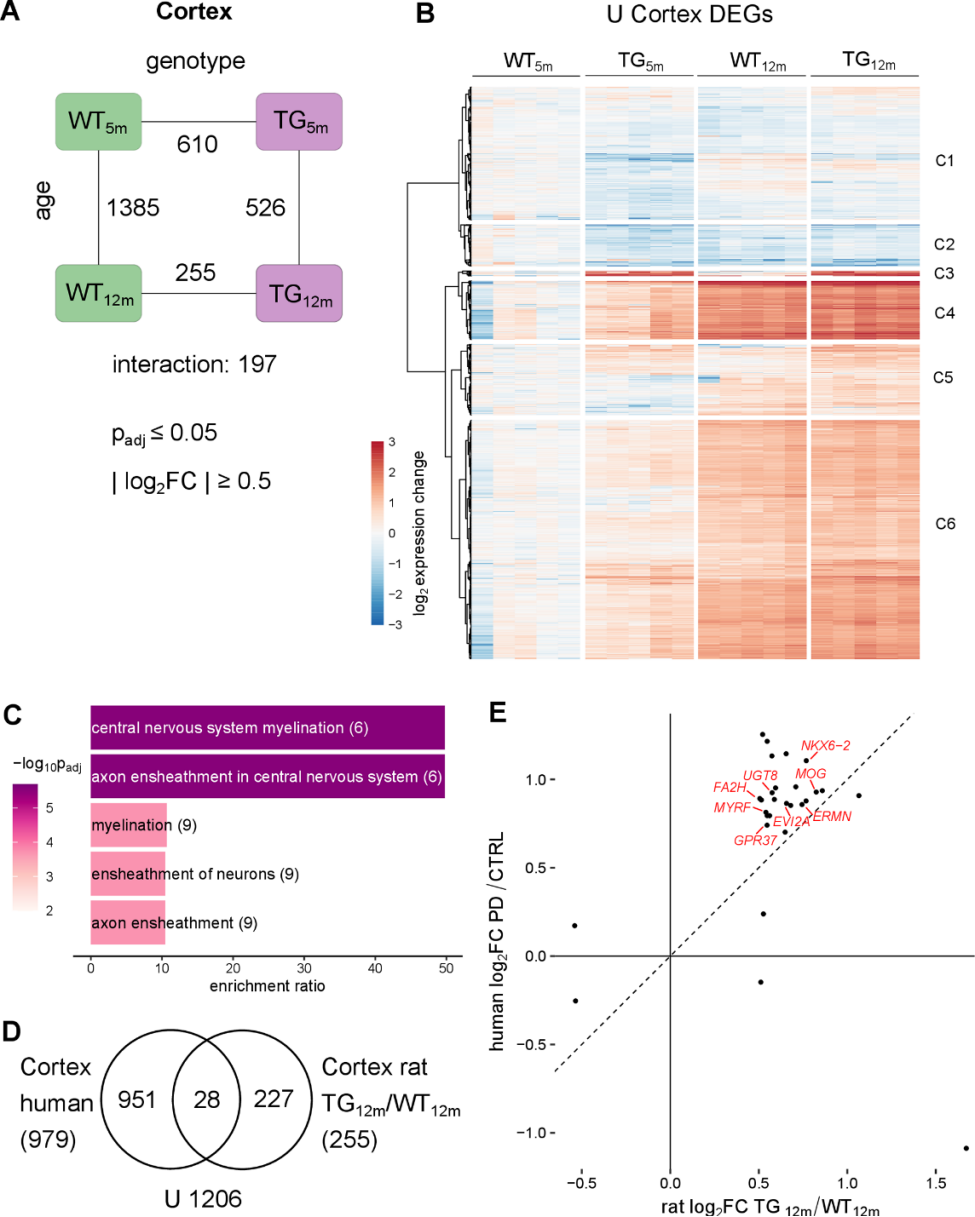
**Cortical transcriptomic perturbations under *SNCA* overload include myelination-linked genes in rat and PD patients.** (**A**) Number of DEGs between experimental groups in the cortex, along the genotype (WT and TG) and age axes (5 and 12 months) and their interaction with the indicated significance cut-offs. (**B**) Heatmap of hierarchically clustered cortical expression changes relative to WT_5m_ of 2061 DEGs (union of cortical DEGs shown in Figure 4A) across experimental groups. (**C**) Five most significant enriched biological processes for the 255 cortical DEGs of 12-month-old TG rats with indicated adjusted p-value, enrichment ratio, and DEG count in brackets. (**D**) Venn diagram comparing 255 DEGs identified in the cortex of 12-month-old TG rats and 979 DEGs identified in the cortex of PD patients [31]. (**E**) Scatter plot of 28 overlapping DEGs identified in the cortex of 12-month-old TG rats and in the cortex of PD patients. Oligodendrocyte associated DEGs are labelled.

In addition, overall higher effect sizes were detected compared to striatum and cerebellum (Figure 4B and Supplementary Figure 8D). Clustering of this expression changes revealed six distinct groups with similar perturbation patterns as seen in the other regions (Figure 4B and Supplementary Figure 12). Also similar to striatum and cerebellum the largest number of DEGs (C4 and C6 in Figure 4B) show the strongest age-dependent upregulation and premature upregulation in young TG animals. This phenomenon was also reflected in the cortical age signature (Supplementary Figure 13). Functionally, the DEGs identified in cluster C4 and C6 were most significantly enriched for biological processes linked to synaptic functions including regulation of postsynaptic membrane potential, regulation of membrane potential, and modulation of chemical synaptic transmission (Supplementary Figure 14A), suggesting that synapse-related pathways are affected early on.

Further exploration of cortical DEGs under *SNCA* overload revealed significant enrichment for central nervous system myelination and axon ensheathment in central nervous system in TG rats, primarily at 12 months of age (Figure 4C and Supplementary Figure 14B). These genes were also significantly enriched for genes attributed to oligodendrocytes (Supplementary Figure 14C), suggesting that myelination-associated and oligodendrocyte-specific genes are significantly perturbed in old TG rats.

To relate these finding in the animal model to human, previously published post-mortem cortical transcriptomes of PD patients were analysed analogous to rat samples [31]. Among 979 genes differentially expressed in the cortex of PD patients, 28 orthologues overlapped with DEGs identified in 12-month-old TG rats (Figure 4D). Intriguingly, 28 shared differential genes are specifically expressed in oligodendrocytes including *MOG*, *UGT8* and *MYRF*, that showed increased and nearly identical expression in old TG rats and patients (Figure 4E). This is in line with previous transcriptomic analyses of cortical samples against other PD patient data [20] but did not extend to protein level (Supplementary Table 2).

Taken together, these results indicate that—compared to striatum and cerebellum—the cortex exhibits the largest effect sizes of age-dependent expression changes. Perturbations under the transgene not only occurred early on but also led to the highest number of genes with significantly different age trajectories under *SNCA* overload. Lastly, upregulation of myelination-associated genes in cortex of TG rats was shared between animal model and PD patients.

### Core set of genes exhibits common perturbation patterns across brain regions in *SNCA* overexpressing rats

**Figure 5:**
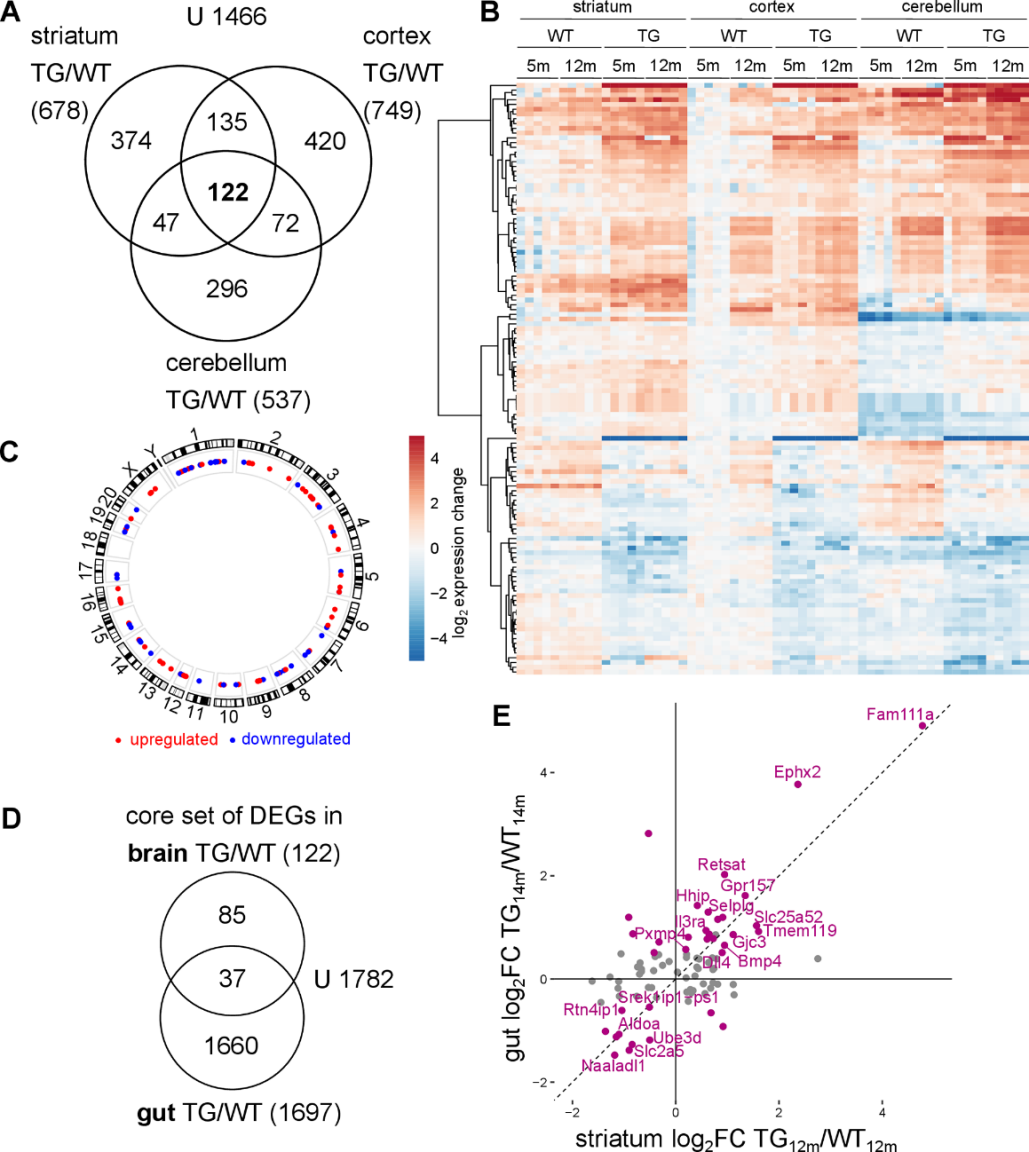
**Common transcriptomic changes in *SNCA* overexpressing rats across brain regions and tissues.** (**A**) Venn diagram comparing DEGs identified along the genotype axis in 5- and 12-month-old WT and TG rats between the striatum, the cortex and the cerebellum. (**B**) Heatmap of striatal, cortical and cerebellar expression profiles of 122 overlapping genes (shown in A) as log_2_ expression change relative to striatal WT_5m_ per experimental group. (**C**) Circos plot of the spatial distribution of 122 common DEGs found in TG animals across all examined brain regions. Genes are color coded based on their up- or downregulation in at least two brain regions. (**D**) Venn diagram comparing 122 DEGs identified as core set of DEGs in three examined brain regions in TG animals and 1697 DEGs identified in the gut of the same rat model [32]. (**E**) Scatter plot of 122 cross-regional DEGs in TG animals in striatum and gut with 37 overlapping DEGs between brain regions and gut highlighted in purple. DEGs with the same regulation in brain and gut are labelled.

To investigate whether the common patterns observed in all examined brain regions are based on the same underlying genes, the overlap of DEGs across brain regions was examined (Figure 5A). Indeed, among the most significant cross-region DEGs was *Sncg*, encoding for gamma-synuclein, another member of the synuclein family, *Ddc* encoding for dopa decarboxylase as well as genes like *Fam111a* and *Gjc3* (Supplementary Figure 15). In addition, there were several cross-region DEGs agreeing with known aSyn biology such as *Orai2* encoding for a calcium channel and *Gpr157*, both showing age-independent upregulation, or *P2rx4* a ligand-gated channel with high calcium permeability significantly downregulated across brain regions in TG animals (Supplementary Figure 15).

Together, the common genes extend to a total of 122 (Figure 5A and Supplementary Table 3). Characterizing them further with respect to pathway and gene set enrichments, and known interactions did not yield significant or conclusive results, emphasizing that these DEGs can be understood only in context of the other region-specific DEGs (Supplementary Figures 3D, 14B). Transcription factor analysis of the 122 genes revealed enrichment of targets of the transcription factor SP1, where SP1 itself was downregulated in an age-dependent manner (Supplementary Figure 16). However, gene regulatory network analysis did not point to specific signalling pathway, even under relaxed criteria (Supplementary Figure 17). Yet, the expression pattern of the core genes is very clear across regions, with largely similar dysregulation in TG animals, and in which most genes show significantly altered expression in both young and old TG animals (Figure 5B). The cross-regional DEGs were also evenly distributed across the genome, suggesting the differential genes not to result from effects in the genomic vicinity of transgene integration sites (Figure 5C).

Further support for non-random effects underlying the core gene set is given by similarities in differential splice events. While the preponderance of them occurs region-specifically, some affected genes are shared across brain regions with nearly identical impact on transcript isoform usage. These overlaps exist specifically with respect to genotype and are exemplified by candidates like *Ptprn*, *Zfyve28*, and *Ifi44* (Supplementary Figure 19).

To investigate whether the core set of differential genes across brain regions are also reflected in another tissue, published transcriptome data from gut epithelial cells of the same rat model [32] were analysed analogous to brain samples. 37 DEGs in the gut overlapped with the core set of differential genes identified in brain (Figure 5D). Again, these common DEGs displayed largely similar expression across both tissues in TG animals (Figure 5E and Supplementary Figure 20), suggesting a systemic effect of *SNCA* overexpression.

These results indicate that although most transcriptomic changes under *SNCA* overexpression are brain region-specific, there is a core set of genes in the brain which share very similar perturbations under aSyn overload. Part of these cross-regional genes are similarly dysregulated in the gut, a tissue known to play an important role in synucleinopathies, particularly in PD [33].

## DISCUSSION

Synucleinopathies share an age component in their etiology and are often not clinically diagnosed before later stages of the pathogenesis, then primarily on motor phenotypes [34]. Due to limited access to human brain, a better molecular understanding of the pathomechanism and its unfolding remains challenging. Animal models can help to address this challenge and lend themselves to longitudinal studies of synuclein biology [35].

Here, a transgenic rat model that overexpressing human *SNCA* on a BAC construct [19] was used to determine gene expression differences in striatal, cortical, and cerebellar tissue of 5- and 12-month-old animals on both gene and transcript level. The expression of the human transgene in this model was closer to physiological levels [19] and, hence, agrees with the overall suitability of these models to mimic the process of slower neurodegeneration in contrast to, for example, toxin-induced models that better capture later aspects of the pathology [36, 37]. However, comparison of transcriptomic changes with other transgenic mouse models of synucleinopathies revealed only limited overlap, which might arise from species-specific differences, brain region- and age-related variability, or distinct molecular contexts inherent to each model system.

Interestingly, no obvious correlation was observed between excess load of *SNCA* and the number of differential genes in the investigated brain regions. In fact, even an age-dependent increase of *SNCA* in striatum resulted in lower DEG count. The magnitude of expression changes over time, however, seemed to be indicative of differential effect sizes in context of the transgene, suggesting that brain regions which undergo stronger expression changes along normal aging also exhibit stronger perturbations under overexpressed *SNCA*. These observations emphasize the need for region- and age-resolved investigations to advance in synuclein biology.

Translating these age- and region-specific gene expression changes to the human condition is typically restricted to only *post-mortem* time points. In addition, not for all brain regions investigated herein human reference data were available. Against this background, comparisons of striatal and cerebral effects between animal model and human remained limited. For cortex, however, previous translational attempts utilizing another human dataset were confirmed and centered on the distinctive upregulation of oligodendrocyte- and myelin-associated genes [20]. These findings are in line with the evolving shift in our understanding of synucleinopathies and include the involvement of non-neuronal cells, in particular myelin-producing oligodendrocytes [38, 39]. Increase of white matter, already in early stages, was observed in PD patients, too, and might facilitate early screenings [40].

Building on the concept of early alterations, the results herein suggest intriguing age-dependent perturbations associated with *SNCA* overexpression. Notably, more pronounced transcriptomic changes occurred in young transgenic animals, particularly in the striatum and cortex. The phenomenon of early alterations aligns with recent studies, suggesting transcriptomic dysregulation arises from an interplay of general brain region-specific vulnerability, age, and *SNCA* overexpression [18, 41–43]. In a BAC-Tg3(SNCA*E46K) transgenic mouse model overexpressing human mutant *SNCA*, the majority of transcriptomic alterations in the ventral midbrain occurred at three months of age [42]. Notably, these changes were detected well before any signs of neurodegeneration. Similarly, in a toxin-induced mouse model of synucleinopathies, in which aSyn preformed fibrils were injected, more pronounced expression changes were detected at the early time point compared to later stages [43]. These early alterations were primarily linked to neuroinflammatory processes, suggesting an active role of microglia in initial stages of the pathology.

In the animal model used herein, the seemingly counterintuitive observation of detecting more expression changes in younger animals than in older can be largely attributed to the premature upregulation of specific genes in young transgenic subjects. This phenomenon represented a dominant and recurring pattern across all brain regions, evident in age-related signature genes and beyond. As animals aged, differences in gene activity between wild type and transgenic animals tended to converge again, resulting in fewer detectable changes by 12 months of age.

Hypothesizing on the biology behind this intriguing pattern, the premature upregulation in transgenic animals might suggest an accelerated aging process [41, 44]. This hypothesis, however, is not fully supported by the data. Crucially, older transgenic animals did not exhibit signs of continued acceleration or exacerbated aging compared to their wild type controls. Moreover, the pathways and cellular functions associated with the genes underlying this expression pattern did not align with typical aging processes. However, definitive conclusions should be approached with caution, as even the 12-month time point is still regarded as middle age in the lifespan of rats [45].

Instead, the pattern suggests a gradual, age-dependent upregulation of certain genes in wild type animals, potentially to adapt to or counteract effects of aging. The pathway enrichments indicate the possibility of physiological or morphological adaptations in synaptic contexts and neuritogenesis, too. In transgenic animals, early activation of the same genes could be compensatory in nature as the system attempts to mitigate the effects of *SNCA* overload [42, 46]. In line, Stemick et al., 2020 report that BAC SNCA rats exhibit neuritogenesis of serotonergic afferents within the dorsal striatum as a compensatory mechanism [47]. Despite severe loss of dopaminergic cells and neurites in the *substantia nigra* and dorsal striatum, these animals exhibit a significant increase in serotonergic fiber density in the dorsal striatum while the number of serotonergic neurons in the raphe nuclei remained unchanged [47]. This suggests that serotonergic plasticity, both at the structural and transcriptomic levels, serves as a compensatory response to *SNCA* overexpression and dopamine depletion in TG animals. Premature upregulation of synaptic genes in young BAC SNCA rats may actively drive serotonergic adaptation, highlighting its potential role in disease progression and compensation of aSyn pathology. Stemick et al., 2020 further identify serotonergic neurons as a potential ectopic source of dopamine in the striatum by expressing key dopaminergic machinery (AADC encoded by *Ddc*, VMAT2 encoded by *Slc18a2*) which led to reduced serotonergic autoreceptor levels (5-HT1A encoded by *Htr1a*, 5-HT1B encoded by *Htr1b*) and excessive dopamine release [47].

In line, the striatal transcriptome presented herein revealed age-dependent expression changes of these key genes in TG animals, with *Ddc* and *Htr1b* showing a higher expression in 5- compared to 12-month-old TG rats, suggesting early transcriptional activation followed by a decline. Although *Slc18a2* was not differentially expressed in TG animals, its premature upregulation in young TG animals aligns with the notion of early serotonergic adaptation in response to *SNCA* overexpression and dopamine depletion. Translating this finding to PD patients, this suggests that early compensatory mechanisms of increased serotonergic sprouting and dopamine release might initially preserve motor function but might also contribute to L-DOPA-induced dyskinesia at later stages. Hypothesizing on the underlying mechanism of early compensatory neuritogenesis, transcription factor analysis further revealed that genes showing early upregulation in TG animals are enriched for targets of Pax6, a regulator of neuronal plasticity [30], suggesting its potential involvement in early serotonergic adaptation. It is important to note that transcriptomic profiling in this study was performed on whole striatal tissue, which consequently might obscure region-specific serotonergic effects reported by Stemick et al., 2020.

The shared expression patterns observed across brain regions led us to investigate common transcriptomic changes arising from the transgene. Indeed, we identified a core set of differential genes affected by *SNCA* overexpression in all examined brain regions. Pure random as well side effects in the vicinity of potential integration sites of the BAC construct seem unlikely given the genomic location of affected genes and highly similar expression pattern in all three brain regions.

Among these cross-regional DEGs neither a specific pathway or cellular function was enriched, nor did a gene regulatory network analysis reveal a dominant regulator or a central signalling pathway, suggesting that the observed transcriptional changes reflect widespread effects of *SNCA* overload, rather than the dysregulation of a singular pathway. However, there were several individual candidates of functional interest, including key genes of the dopaminergic machinery like *Ddc* [48], as well as *Sncg* encoding for gamma-synuclein, another member of the synuclein family [49], which can induce aggregations of aSyn and can aggregate itself. Increased expression of *Sncg* was also described in a toxin-induced non-human primate model of PD [50]. Among the cross-regional DEGs were several calcium-channels previously linked to PD including *Orai2*, *Gpr157*, and *P2rx4* [51–53]. The core set of genes also included *Fam111a*, one of the highest overexpressed gene across all brain regions, encoding a serin protease involved in DNA replication and antiviral defence, whose protease activity is linked to apoptosis [54, 55]. Transcriptomic analysis of a toxin-induced rat model of PD identified *Fam111a* as differentially expressed across five brain regions, linked to synaptic dysfunction [56]. However, the mechanistic role of *Fam111a* in PD pathogenesis remains unclear. Transcription factor analysis revealed enrichment for target genes of SP1 among the cross-regional DEGs, with SP1 activity being reduced in an age-dependent manner. Downregulation of SP1 was shown to be neuroprotective in a toxin induced mouse model of PD [57], therefore its initial downregulation may reflect a compensatory response to *SNCA* overexpression in young TG animals.

Notably, many of the common genes across brain regions were recovered even in gut tissue, pointing to a region-independent regulatory core upon which *SNCA*-induced perturbations unfold in a region-specific manner. Although specific pathological changes along the gut-brain axis have not yet been characterized in BAC SNCA rats, prior studies in BAC SNCA mice reported phosphorylated alpha-synuclein in the dorsal motor nucleus of the vagus nerve as early as 2 months of age, which coincided with significant gait alterations in young TG animals [58]. These findings highlight a potential link between early peripheral pathology and central motor symptoms. Furthermore, previous work has demonstrated altered microbiome composition and alpha-synuclein protein expression in the gut of 2-months-old BAC SNCA rats, with progressive accumulation of alpha-synuclein in the gut over time [32]. These peripheral changes support the idea that gut-derived alpha-synuclein may play a role in shaping the disease phenotype [59]. The distinctive pattern of gene expression changes across diverse tissues suggests a fundamental, systemic response to *SNCA* overexpression. It implies that while the core regulatory response may be consistent across tissues, the specific manifestations and downstream effects vary depending on the cellular context and function. Although the shared DEGs do not converge on a single pathway, they comprise multiple cellular functions aligning with known PD-related processes including oxidative stress response and proteostasis (*Ephx2*, *Rtn4ip1*, *Retsat*, *Pex11a*, *Ube3d*) or immune signalling (*Ilr3a*, *Selplg*, *Mpeg1*, *Tmem119*), the latter being consistent with an activated inflammatory environment in the gut of BAC SNCA rats [32]. While exact mechanistic pathways remain speculative, the observed transcriptomic changes in both brain and gut align with the hypothesis of an early, gut-brain interaction in PD pathophysiology.

While it remains to future studies to examine the consequences of these transcriptomic changes on protein level and advance to functional implications, the genes and patterns we highlight here might serve as anker points to direct efforts to. In particular, the involvement of serotonergic adaptions—highlighted both on transcriptome level and in previous studies—points to serotonergic receptor modulators as a promising avenue for future investigation, especially in the context of L-DOPA-induced dyskinesia. Pharmacological targeting of these pathways may help clarify the dual role of serotonergic plasticity as both compensatory and maladaptive in synucleinopathies.

## MATERIALS AND METHODS

### Experimental animals and tissue preparation

Male homozygous transgenic rats overexpressing full-length human *SNCA* including its regulatory elements [19] as well as WT rats with the same genetic background (Sprague-Dawley) were housed in a standard environment until the age of 5 and 12 months. Experimental animals were obtained by crossing heterozygous male with heterozygous female rats. Homozygous or WT status was confirmed by genotyping with quantitative PCR using DNA from ear biopsies with primer sequences specifically for human *SNCA* (F: 5′ccgctcgagcggtaggaccgcttgttttagac-3′; R: 5′-ctctttccacg ccactatc-3′) and normalized to β-actin as reference (F: 5′agccatgtacgtagccatcca-3′; R: 5′-tctccggagtccatcacaatg-3′). Animals were anaesthetized, decapitated, and the brain immediately dissected on ice and snap frozen in liquid nitrogen. The tissue was stabilized with RNAlater (Qiagen) at 4° C.

### RNA isolation and sequencing

The polyadenylated fraction of RNA isolated from striatum, frontal cortex and cerebellum (n = 5 animals in each of the four experimental groups per brain region) was used for RNA-seq. Total RNA, miRNA, and DNA were simultaneously extracted using the DNA/RNA/microRNA Universal Kit (Qiagen) using the manufacturer protocol. Quality was assessed with an Agilent 2100 Bioanalyzer. Samples with high RNA integrity number (RIN > 7) were selected for library construction. Using the TruSeq RNA Sample Prep Kit (Illumina) and 100 ng of total RNA for each sequencing library, poly(A) selected paired-end sequencing libraries (125 bp read length) were generated according to manufacturer’s instructions. All libraries were sequenced on an Illumina HiSeq 2500 platform at a depth of 20–35 million reads each.

### Quality control, alignment and expression analysis

The Nf-core RNA-seq Pipeline (v3.13.0) [24] served as the primary tool for processing the sequencing data. To assess the read quality of the RNA-seq data FastQC (v0.12.0) was used [60]. Reads were aligned against a custom-built reference genome of the Ensembl *Rattus norvegicus* genome (mRatBN7.2) including the human *SNCA* transgene using STAR [61]. Normalized read counts were obtained with Rsubread (v2.12.3) [62]. Quality control metrics from all samples were aggregated and reviewed using MultiQC (v1.14) [63], including e.g. total number of mapped reads, read coverage, duplication rates, and read distribution across genomic features, to ensure consistency across samples. To confirm the absence of outlier samples, principal component analysis (PCA) was performed on normalized gene expression data. All samples clustered with their respective experimental groups, and no outliers were identified.

DESeq2 (v1.38.3) [64] was used for the differential gene expression analysis. Transcripts with less than 20 reads in the median of all samples were excluded from subsequent differential analysis resulting in 14,948 genes for striatum, 14,697 for cortex and 14,521 for cerebellum. Gene expression was modeled in a 2 x 2 factorial design as a function of genotype, age and their interaction Supplementary Tables 4–6. Significance thresholds for differentially expressed genes were set to a BH-adjusted p-value ≤ 0.05 and a |log2FC| ≥ 0.5. Surrogate variable analysis (v3.46.0) [65] was applied to remove unwanted variation in the data.

Transcript abundance was quantified with Salmon (v1.10.1, parameters: numGibbsSamples 20, seqBias, gcBias, validateMappings) [25] using a gentrome based on the Ensembl *Rattus norvegicus* genome and transcriptome including human *SNCA*. Transcripts per million (TPM) values were imported and scaled (scaleInfReps) with Tximeta (v1.16.1) [66]. Only transcripts with at least 50 counts in at least 5 samples were considered for the differential transcript usage analysis. For differential splicing analysis rMATs (v4.3.0) [67] was used with default parameters. To identify significant splicing events filtering of detected splice events was based on ∆PSI ≥ 0.1 (Percent Spliced In) and a BH-adjusted p-value ≤ 0.01. Sashimi plots to visualize differential splicing events were generated using ggsashimi [68].

Heatmaps and centroids were plotted as log_2_ expression changes relative to the mean expression of WT_5m_ samples. nRPKM values (normalized Reads Per Kilobase per Million total reads) were calculated using read counts from DESeq2 to measure the relative gene expression changes [69]. For gene ontology analysis WebGestalt and gProfiler [70] were used to identify overrepresented biological processes for differentially expressed genes [71]. Gene set enrichment analysis was conducted using FGSEA [72]. Transcription factor prediction was carried out using decoupleR [73]. Gene regulatory network analysis was performed using Neko [74] in conjunction with the Omnipath interaction database [75]. Bimodal interactions were excluded from the analysis and intermediated nodes were introduced to capture potential regulatory signalling pathways by setting the indirect network pathway length to one.

### Data availability

RNA-seq data files have been uploaded to GEO database and are available under the accession number GSE281984.

## Supplementary Material

Supplementary Figures

Supplementary Table 1

Supplementary Table 2

Supplementary Table 3

Supplementary Table 4

Supplementary Table 5

Supplementary Table 6
