# Supplementary material for: Brain region-specific and systemic transcriptomic alterations in a human alpha-synuclein overexpressing rat model
Source: Aging (Albany NY). 2025 Oct 20;17(10):2598–636. doi: 10.18632/aging.206331 (PMC12606970; doi:10.18632/aging.206331)
Supplement: Supplementary Figures [file aging-17-10-206331-s001.pdf]

SUPPLEMENTARY FIGURES

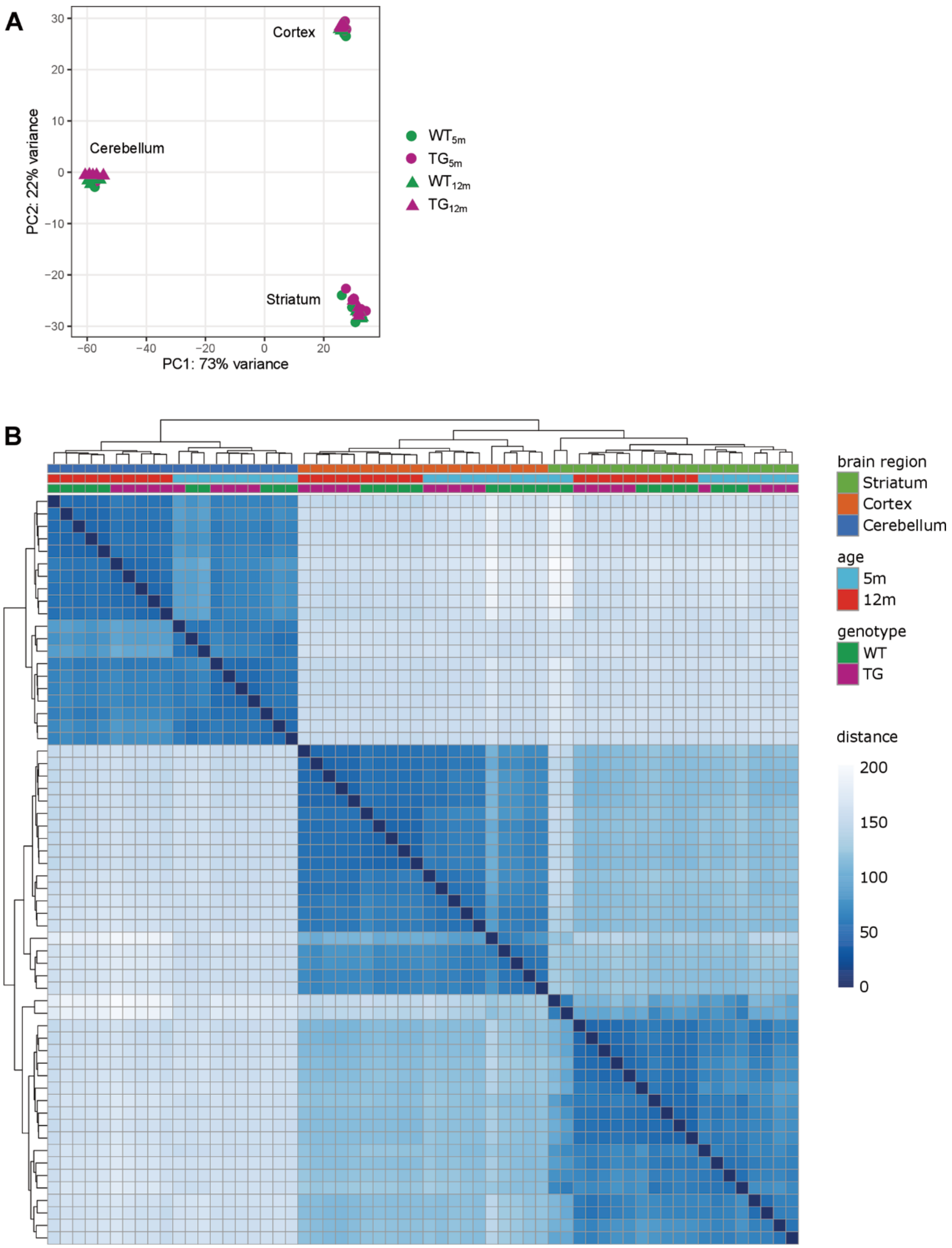

Supplementary Figure 1. Samples are separated by brain region, age, and genotype. (**A**) Principal component analysis of the top 500 most variable genes for all samples. The percentages along the axes represent variance explained between samples for first and second principal component. (**B**) Heatmap of sample-to-sample distance. Gradient of blue shows Euclidean distance and samples are color-coded based on experimental group.

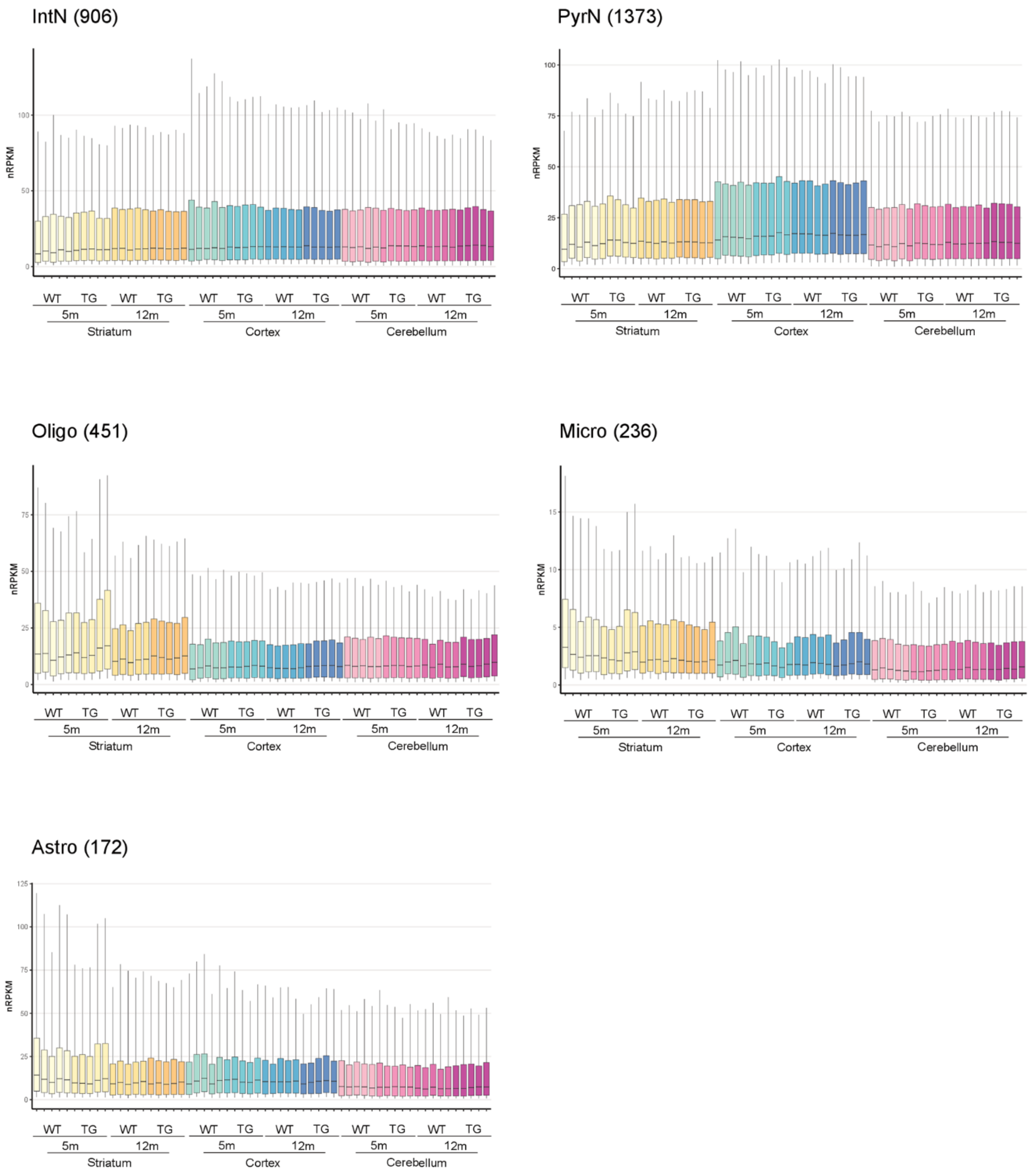

Supplementary Figure 2. Cell type composition estimates between striatal, cortical, and cerebellar samples. Cell type-specific gene expression for five reference cell types in 5- and 12-month-old WT and TG rats per brain region. Expression for each cell type based on reference single-cell data [26], shown as boxplots with geometric mean as well as 10th, 25th, 75th, and 90th quantile. Number of considered genes in brackets for interneurons (IntN), pyramidal neurons (PyrN), oligodendrocytes (Oligo), microglia (Micro) and astrocytes (Astro).

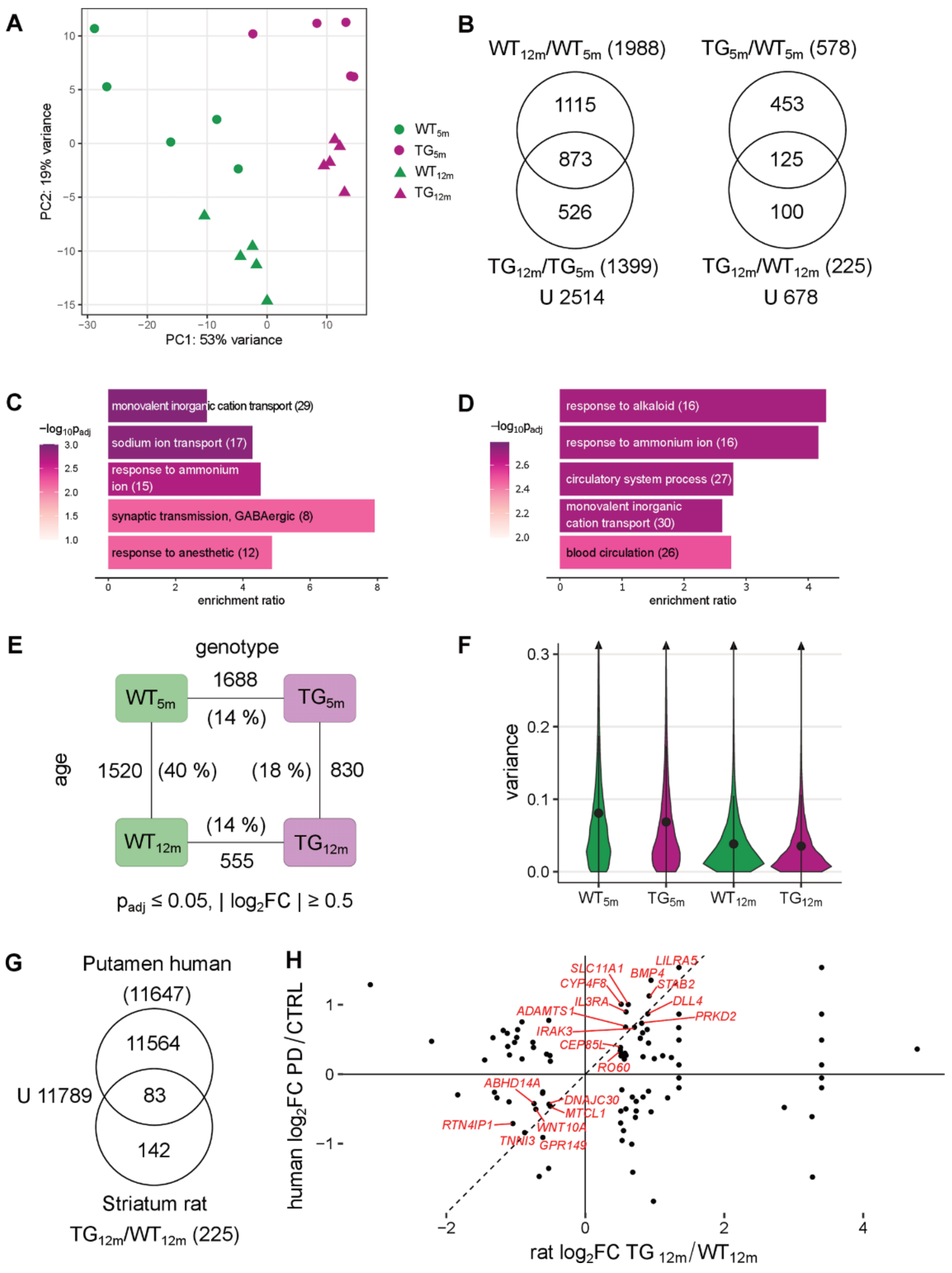

Supplementary Figure 3. Age-dependent transcriptomic changes in the striatum of *SNCA* overexpressing rats. (**A**) Principal component analysis of top 500 most variable genes in striatum for five rat samples per experimental group. The percentages along the axes represent variance explained between groups for first and second principal component. (**B**) Venn diagram comparing DEGs identified along the age axis in WT and TG rats between 5 and 12 months of age (left) and along the genotype axis between 5- and 12-month-old TG rats (right) in the striatum. (**C**) Five most significant enriched biological processes for the 578 DEGs in 5-month-old TG rats (shown in Figure 2A) with indicated adjusted p-value, enrichment ratio and DEG count in brackets. (**D**) Five most significant enriched biological processes for 678 striatal DEGs in 5- and 12-month-old TG animals (shown in Figure 5A) with indicated adjusted p-value, enrichment ratio and DEG count in brackets. (**E**) Number of differential transcripts between experimental groups in striatum, along the genotype (WT and TG) and age axes (5 and 12 months) with the proportion of overlapping corresponding DEGs in brackets and the indicated significance cut-offs. (**F**) Violin plot showing the distribution of gene-wise expression variance (calculated from variance-stabilized transformed expression values) for experimental groups for striatum with mean and standard error of the mean. Y-axis was limited to a maximum of 0.3 for better visualization of group differences and small triangle indicates presence of few outlier genes with higher variance values (up to ~ 6). (**G**) Venn diagram comparing 225 DEGs identified in the striatum of 12-month-old TG rats and 11647 DEGs identified in the putamen of PD patients [28]. (**H**) Scatter plot of 83 overlapping DEGs identified in the striatum of 12-month-old TG rats and in the putamen of PD patients.

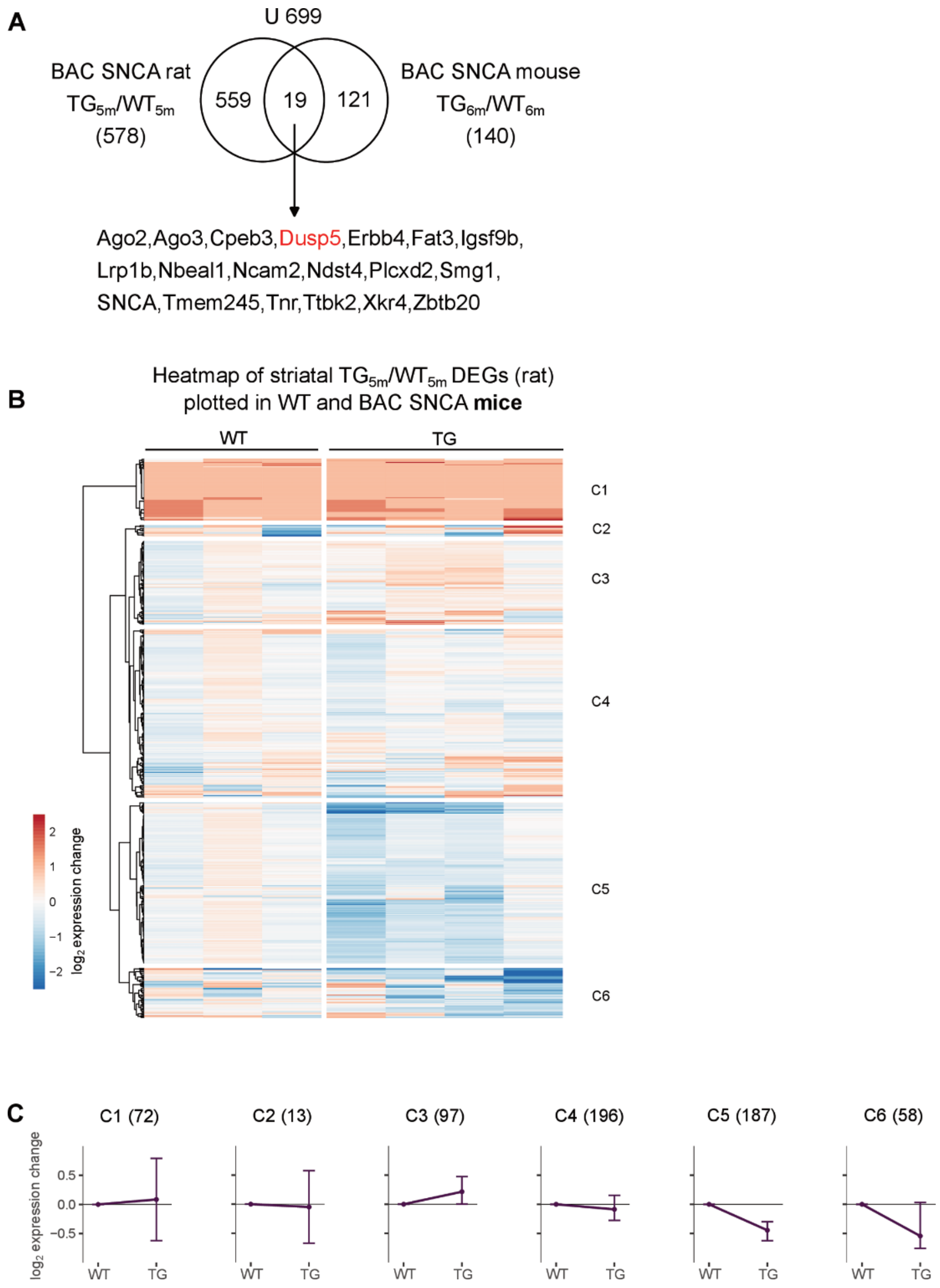

Supplementary Figure 4. Comparison of striatal gene expression changes in young BAC SNCA rats and young BAC SNCA mice. (**A**) Venn diagram comparing DEGs identified in the striatum of 5-month-old BAC SNCA rats and DEGs identified in the striatum of 6-month-old BAC SNCA mice. Overlapping genes with same regulation are marked in red. (**B**) Heatmap of hierarchically clustered striatal expression changes of DEGs identified in 5-month-old TG rats plotted in 6-month-old WT and BAC SNCA mice as log_2 expression change relative to WT samples. (**C**) Average gene expression changes and standard deviation in the striatum of WT and BAC SNCA mice for DEGs identified in TG rats, plotted as centroids clustered in six groups. Numbers of DEGs are shown in brackets.

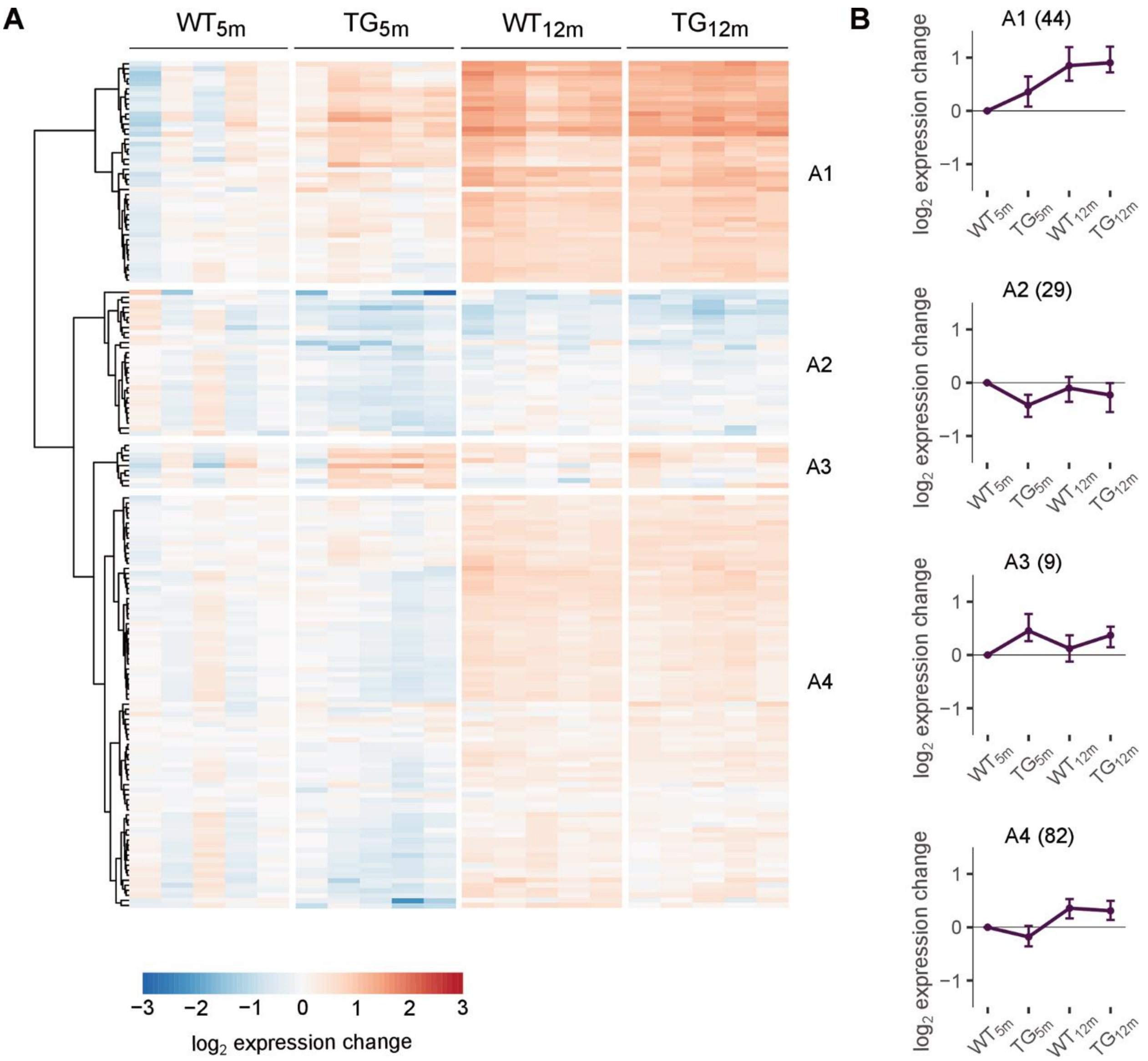

Supplementary Figure 5. Gene expression profile of a striatum-specific age signature. (**A**) Heatmap of hierarchically clustered striatal expression profiles of age-dependent differentially expressed reference genes [23] as $\log_2$ expression change relative to WT_{5m} per experimental group. (**B**) Average gene expression changes and standard deviation plotted as centroids for age-dependent reference DEGs clustered in four groups. Numbers of genes shown in brackets.

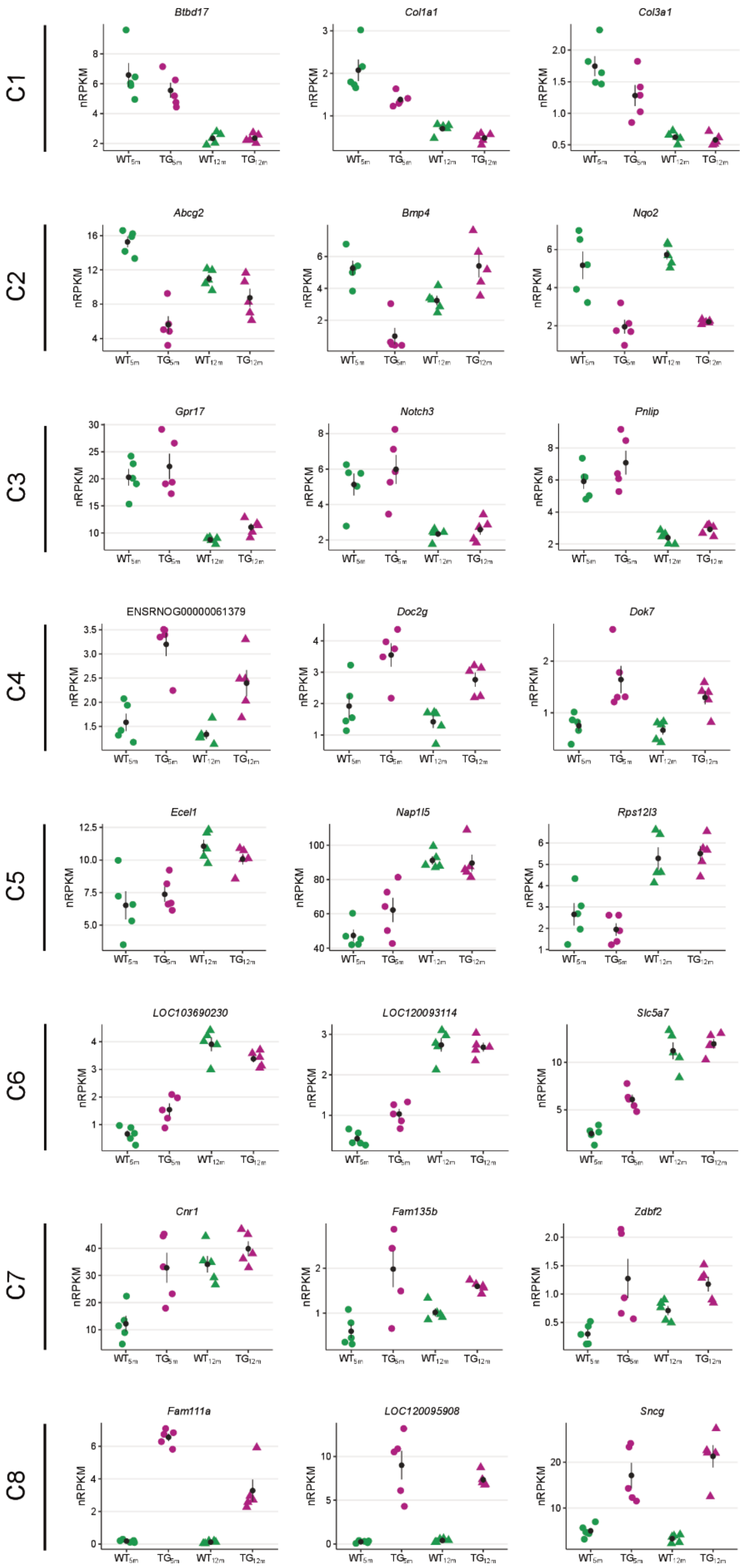

Supplementary Figure 6. Expression level of top three ranked genes of hierarchically clustered striatal expression changes. Top three ranked genes per cluster of Figure 2B with individual nRPKM data points per rat across experimental groups with mean and standard error of the mean. Genes were selected based on the combined rank of log_2FC and p_{adj} from the differential comparison with the highest average absolute expression change for each cluster.

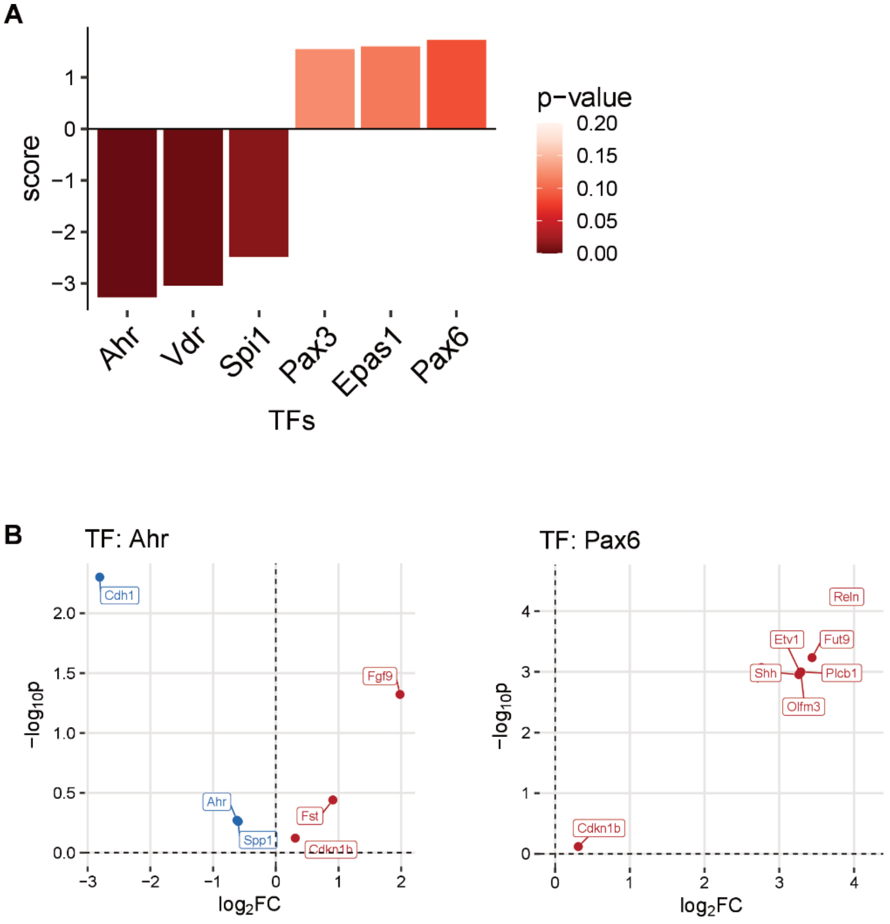

Supplementary Figure 7. Transcription factor activity of premature upregulated genes in the striatum of *SNCA* overexpressing rats. (**A**) Transcription factor activity for the top three most active and inactive transcription factors with activity score and p-value. (**B**) Volcano plot of the differential target genes of the most active and inactive transcription factors for the young TG rats. Genes marked in blue deactivate the transcription factor and genes marked in red activate the transcription factor.

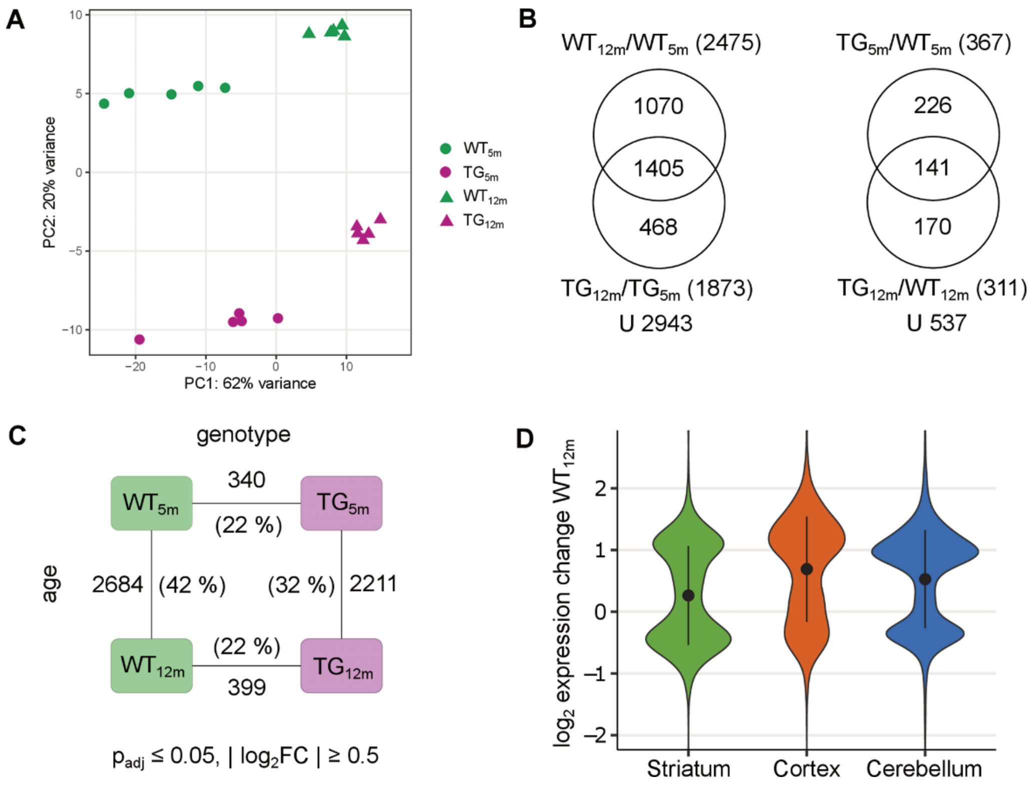

Supplementary Figure 8. Age-dependent transcriptomic changes in the cerebellum of *SNCA* overexpressing rats. (**A**) Principal component analysis of top 500 most variable genes in cerebellum for five rat samples per experimental group. The percentages along the axes represent variance explained between groups for the first and second principal component. (**B**) Venn diagram comparing DEGs identified along the age axis in WT and TG rats between 5 and 12 months of age (left) and along the genotype axis between 5- and 12-month-old TG rats (right) in the cerebellum. (**C**) Number of differential transcripts between experimental groups in cerebellum, along the genotype (WT and TG) and age axes (5 and 12 months) with the proportion of overlapping corresponding DEGs in brackets and indicated significance cut-offs. (**D**) Violin plot showing distribution of expression changes of union DEGs from WT_{12m} samples as log_2 expression change relative to WT_{5m} across brain regions with mean and standard error of the mean.

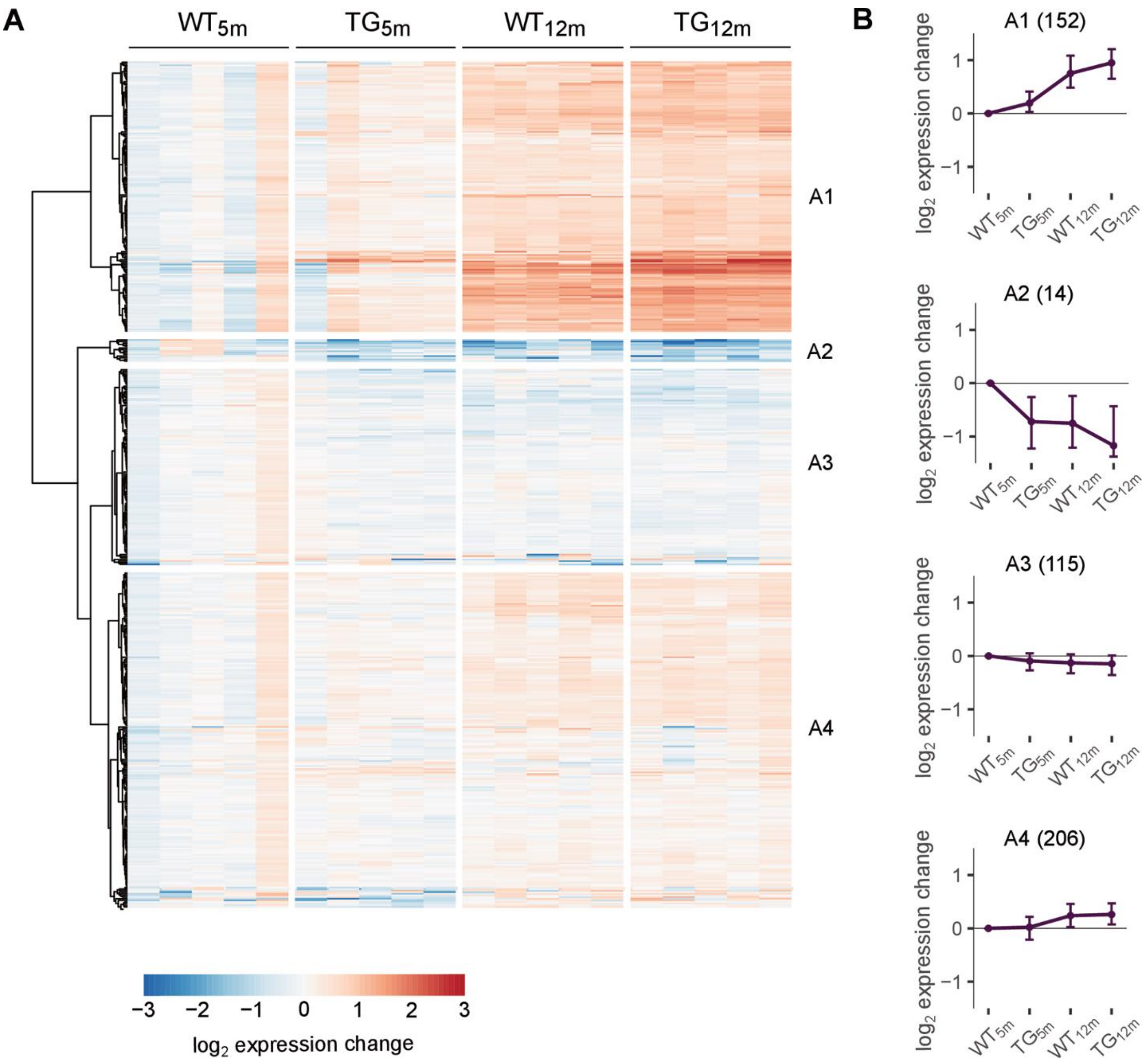

Supplementary Figure 9. Gene expression profile of a cerebellum-specific age signature. (**A**) Heatmap of hierarchically clustered cerebellar expression profiles of age-dependent differentially expressed reference genes [23] as log_2 expression change relative to WT_{5m} per experimental group. (**B**) Average gene expression changes and standard deviation plotted as centroids for age-dependent reference DEGs clustered in four groups. Numbers of genes are shown in brackets.

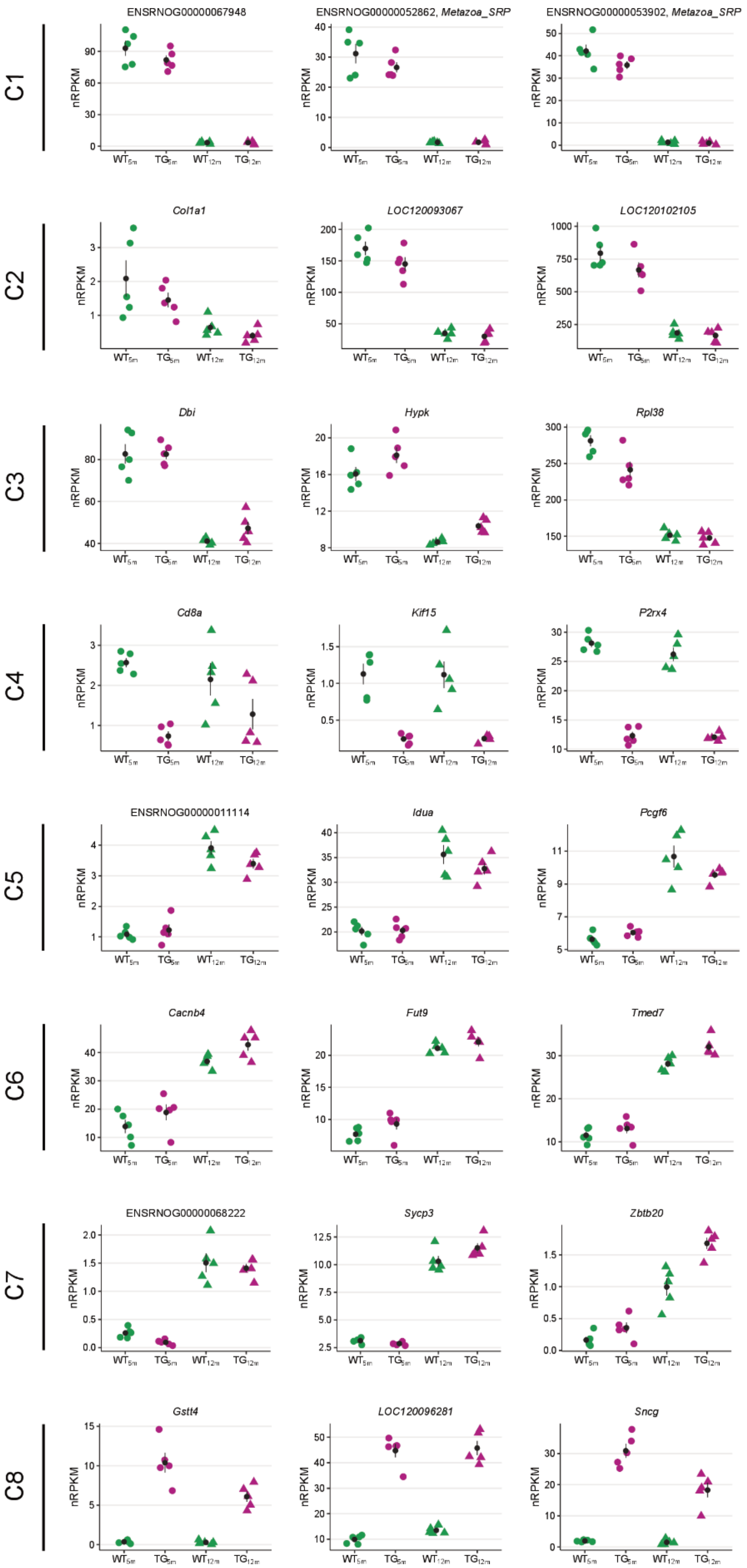

Supplementary Figure 10. Expression level of top three ranked genes of hierarchically clustered cerebellar expression changes. Top three ranked genes per cluster of Figure 3B with individual nRPKM data points per rat across experimental groups with mean and standard error of the mean. Genes were selected based on the combined rank of log_2FC and p_{adj} from the differential comparison with the highest average absolute expression change for each cluster.

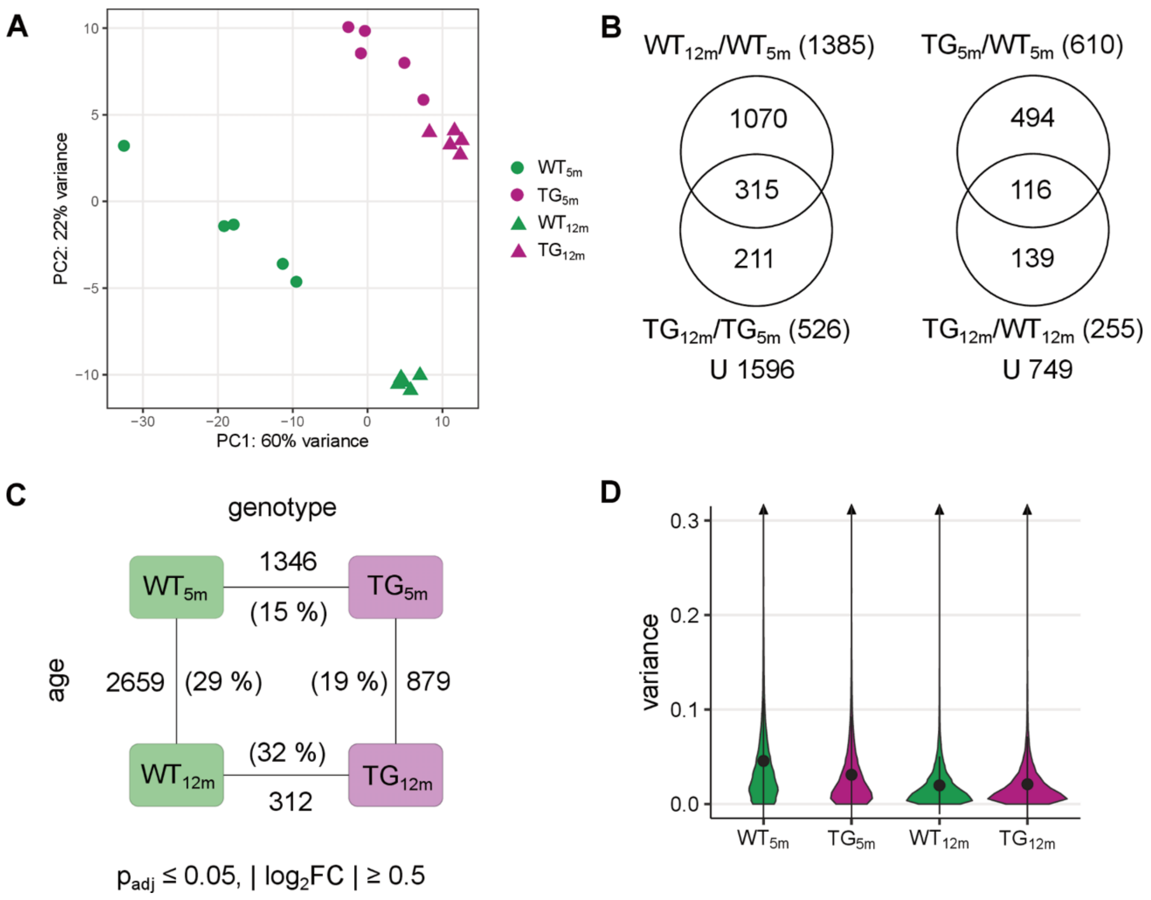

Supplementary Figure 11. Age-dependent transcriptomic changes in the cortex of *SNCA* overexpressing rats. (**A**) Principal component analysis of top 500 most variable genes in cortex for five rat samples per experimental group. The percentages along the axes represent the variance explained between groups for first and second principal component. (**B**) Venn diagram comparing DEGs identified along the age axis in WT and TG rats between 5 and 12 months of age (left) and along the genotype axis between 5- and 12-month-old TG rats (right) in the cortex. (**C**) Number of differential transcripts between experimental groups cortex, along the genotype (WT and TG) and age axes (5 and 12 months) with the proportion of overlapping corresponding DEGs in brackets and the indicated significance cut-offs. (**D**) Violin plot showing the distribution of gene-wise expression variance (calculated from variance-stabilized transformed expression values) for experimental groups for cortex with mean and standard error of the mean. Y-axis was limited to a maximum of 0.3 for better visualization of group differences and small triangle indicates presence of few outlier genes with higher variance values (up to ~ 3).

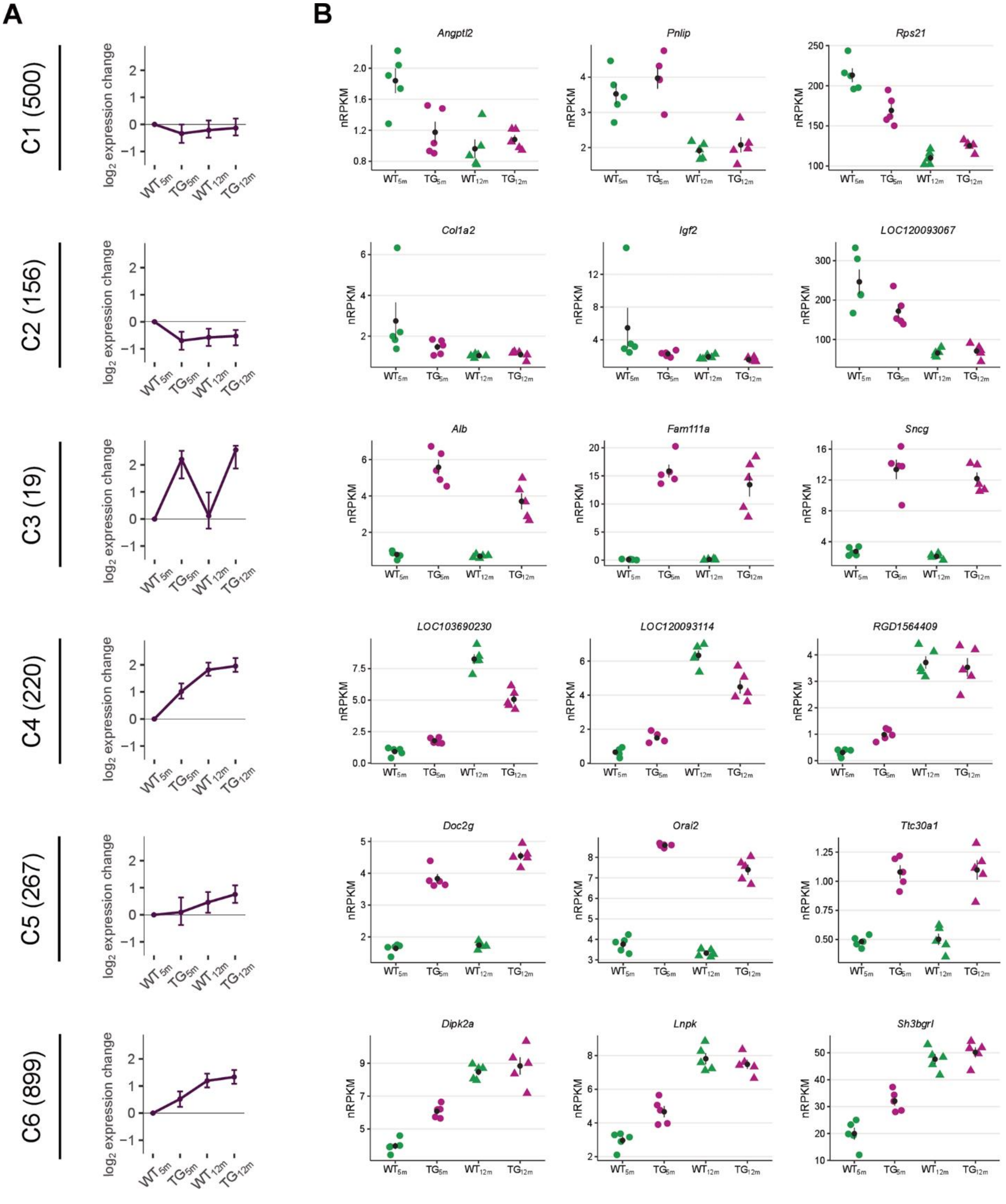

Supplementary Figure 12. Gene expression profiles of hierarchically clustered cortical expression changes. (**A**) Average gene expression changes and standard deviation of all cortical DEGs plotted as centroids clustered in eight groups. Numbers of DEGs are shown in brackets. (**B**) Expression level of top three ranked genes per cluster with individual nRPKM data points per rat across experimental groups with mean and standard error of the mean. Genes were selected based on the combined rank of log_2FC and p_{adj} from the differential comparison with the highest average absolute expression change for each cluster.

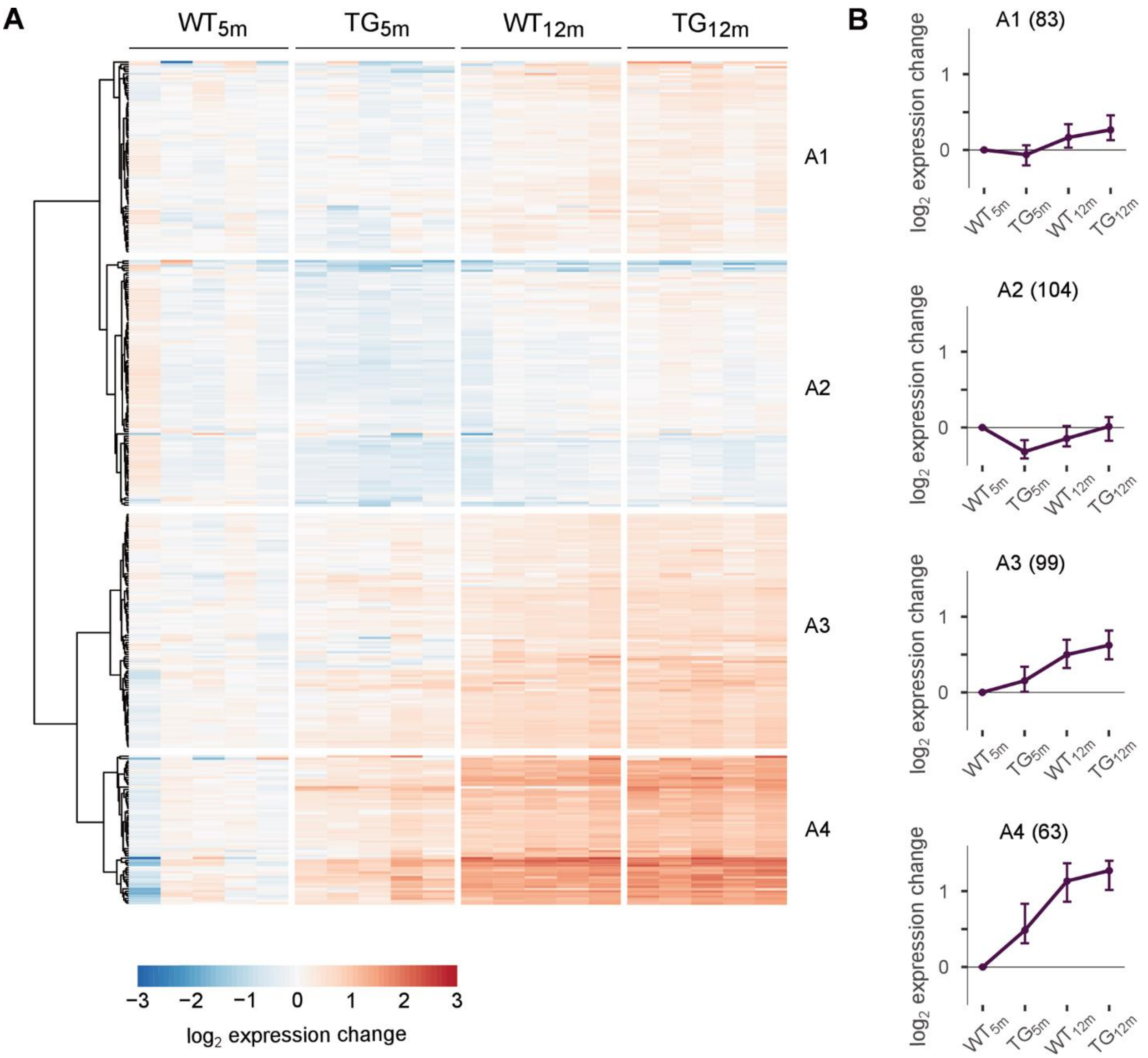

Supplementary Figure 13. Gene expression profile of a cortex-specific age signature. (**A**) Heatmap of hierarchically clustered cortical expression profiles of age-dependent differentially expressed reference genes [23] as log_2 expression change relative to WT_{5m} per experimental group. (**B**) Average gene expression changes and standard deviation plotted as centroids for age-dependent reference DEGs clustered in four groups. Numbers of genes in brackets.

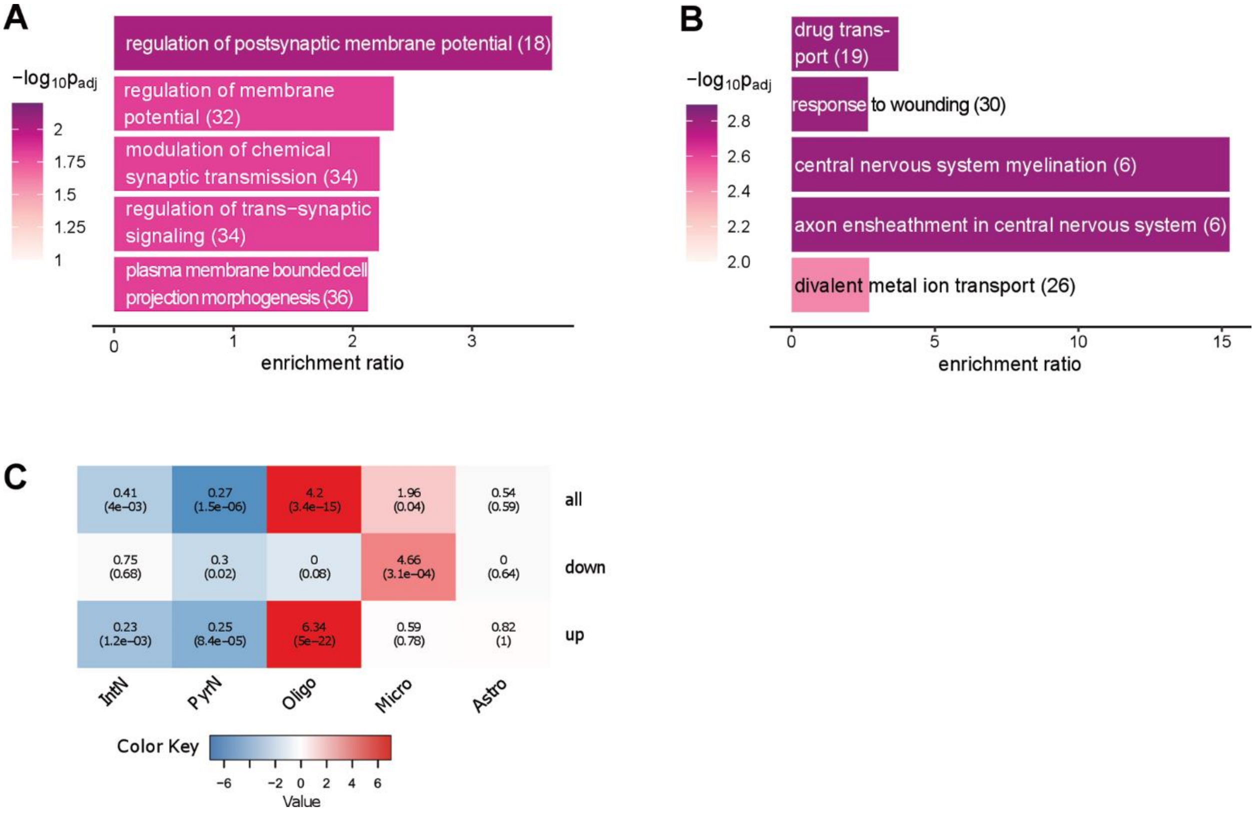

Supplementary Figure 14. Cortical gene expression changes in *SNCA* overexpressing rats. (**A**) Five most significant enriched biological processes for the DEGs in Cluster C4 and C6 with indicated adjusted p-value, enrichment ratio and DEG count in brackets. (**B**) Five most significant enriched biological processes for 749 cortical DEGs in 5- and 12-month-old TG animals (shown in Figure 5A) with indicated adjusted p-value, enrichment ratio and DEG count in brackets. (**C**) Cell type enrichment analysis of 255 DEGs identified in the cortex of 12-month-old TG rats. Shown is the fold enrichment for genes attributed to interneurons (IntN), pyramidal neurons (PyrN), oligodendrocytes (Oligo), microglia (Micro), and astrocytes (Astro) [26]. p-value represents significance in enrichment (red) or depletion (blue) over background by two-sided Fisher's exact test.

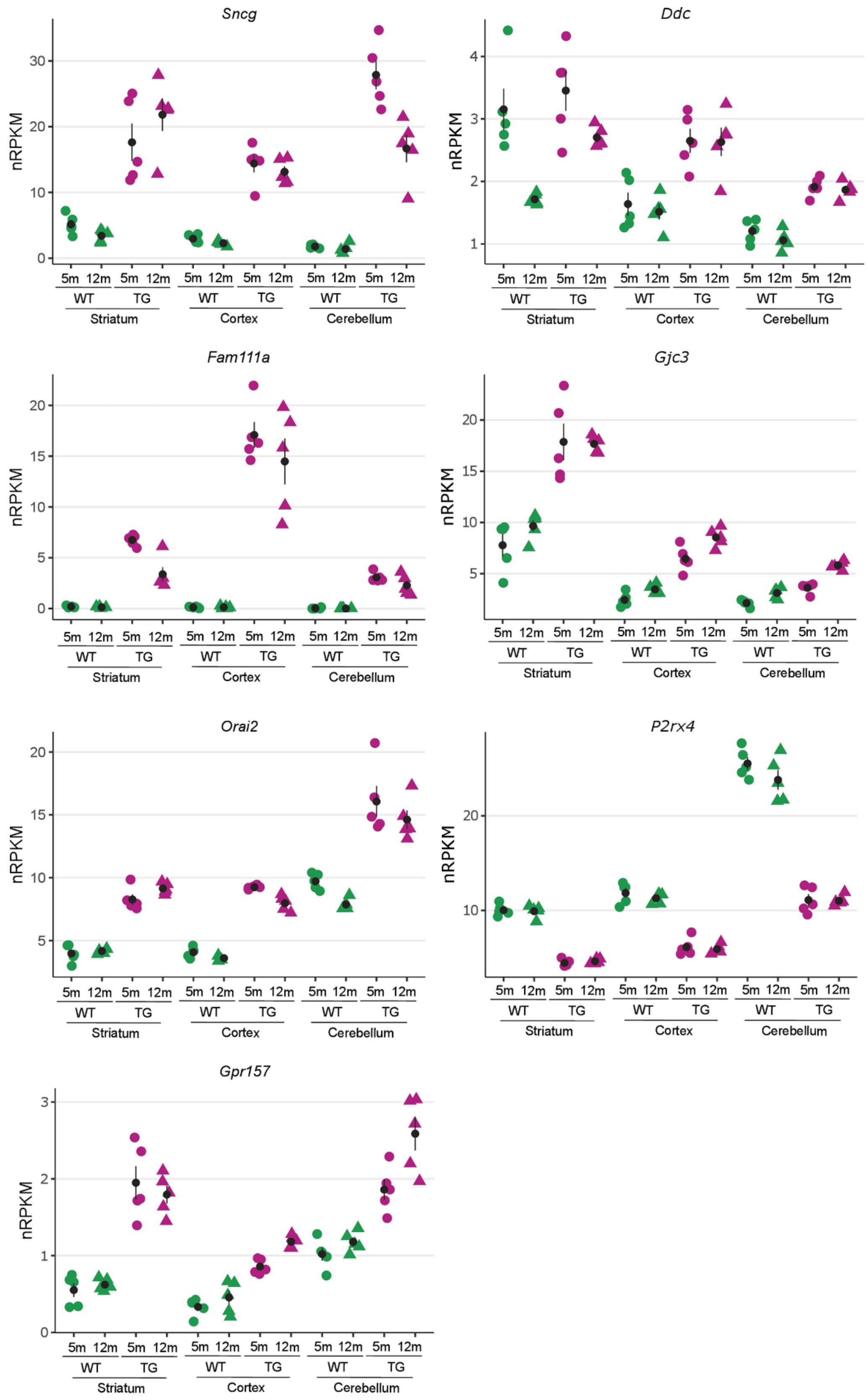

Supplementary Figure 15. Similar dysregulated genes in *SNCA* overexpressing rats across brain regions. Expression level of *Fam111a*, *Gjc3*, *Gpr157*, *Sncg*, *Ddc*, *Orai2*, and *P2rx4* as individual nRPKM data points per rat across experimental groups with mean and standard error of the mean.

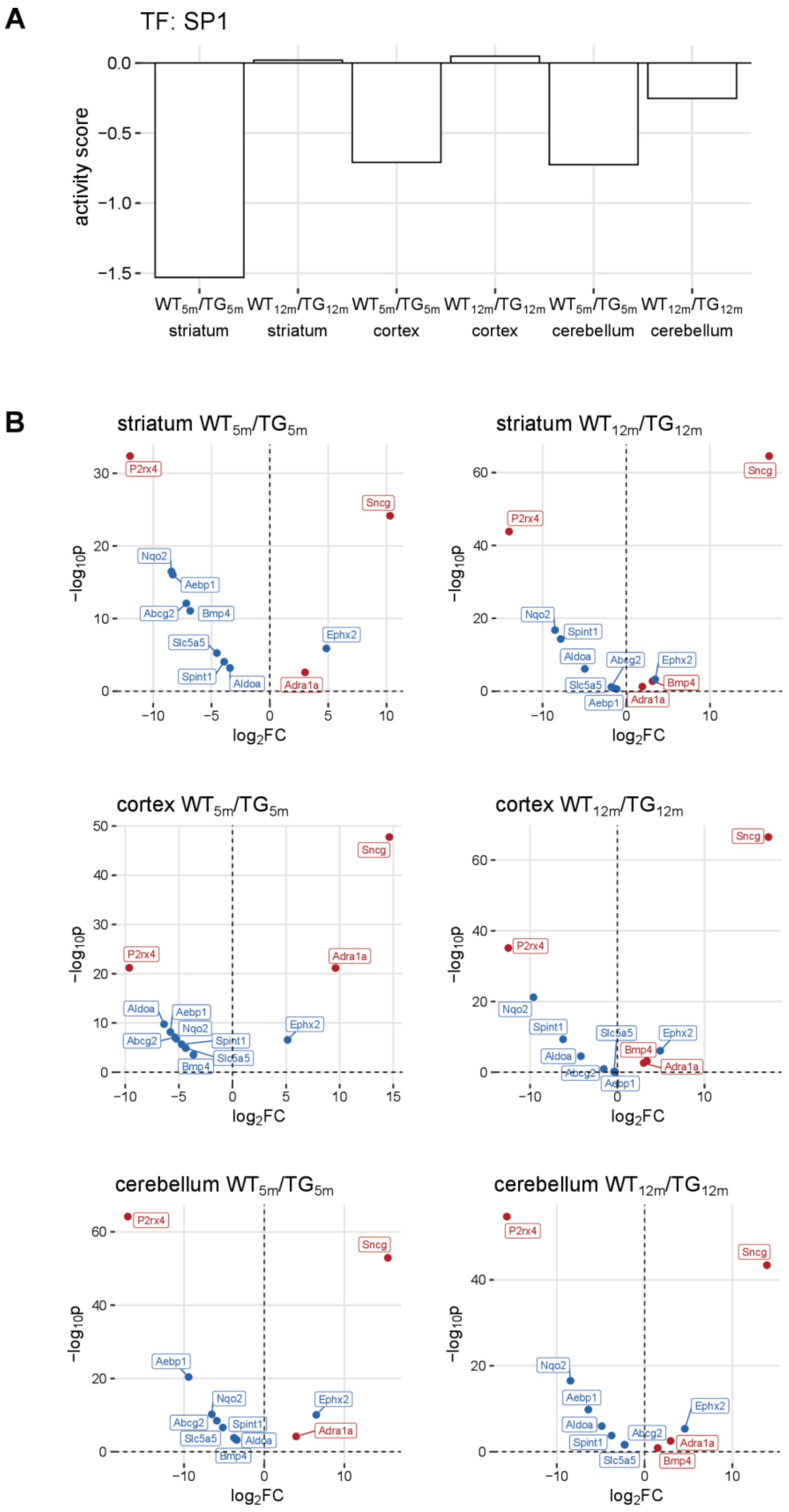

Supplementary Figure 16. SP1 transcription factor activity across brain regions of *SNCA* overexpressing rats. (**A**) SP1 transcription factor activity with the activity score across brain regions in 5- and 12-month-old TG rats. (**B**) Volcano plot of SP1 differential target genes across contrasts. Red and blue genes activate and deactivate SP1, respectively.

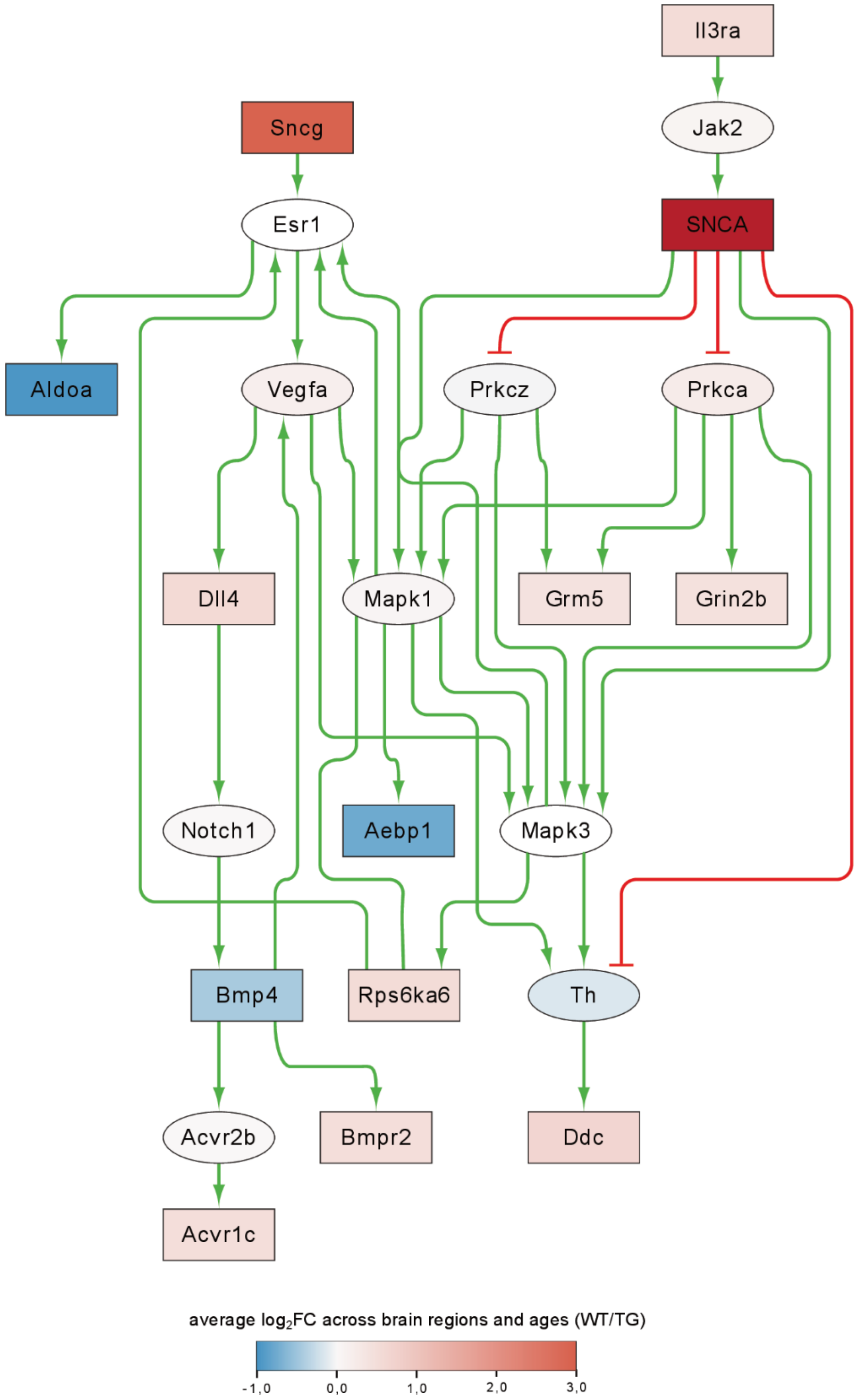

Supplementary Figure 17. Gene regulatory network of the core set of differentially expressed genes across brain regions in *SNCA* overexpressing rats. Squares represent DEGs that are part of the core set, circles represent intermediate nodes not part of the core set. Green edges represent activating interactions, red edges inhibitory. Node color encodes average gene expression $\log_2$ fold-change across brain regions and ages.

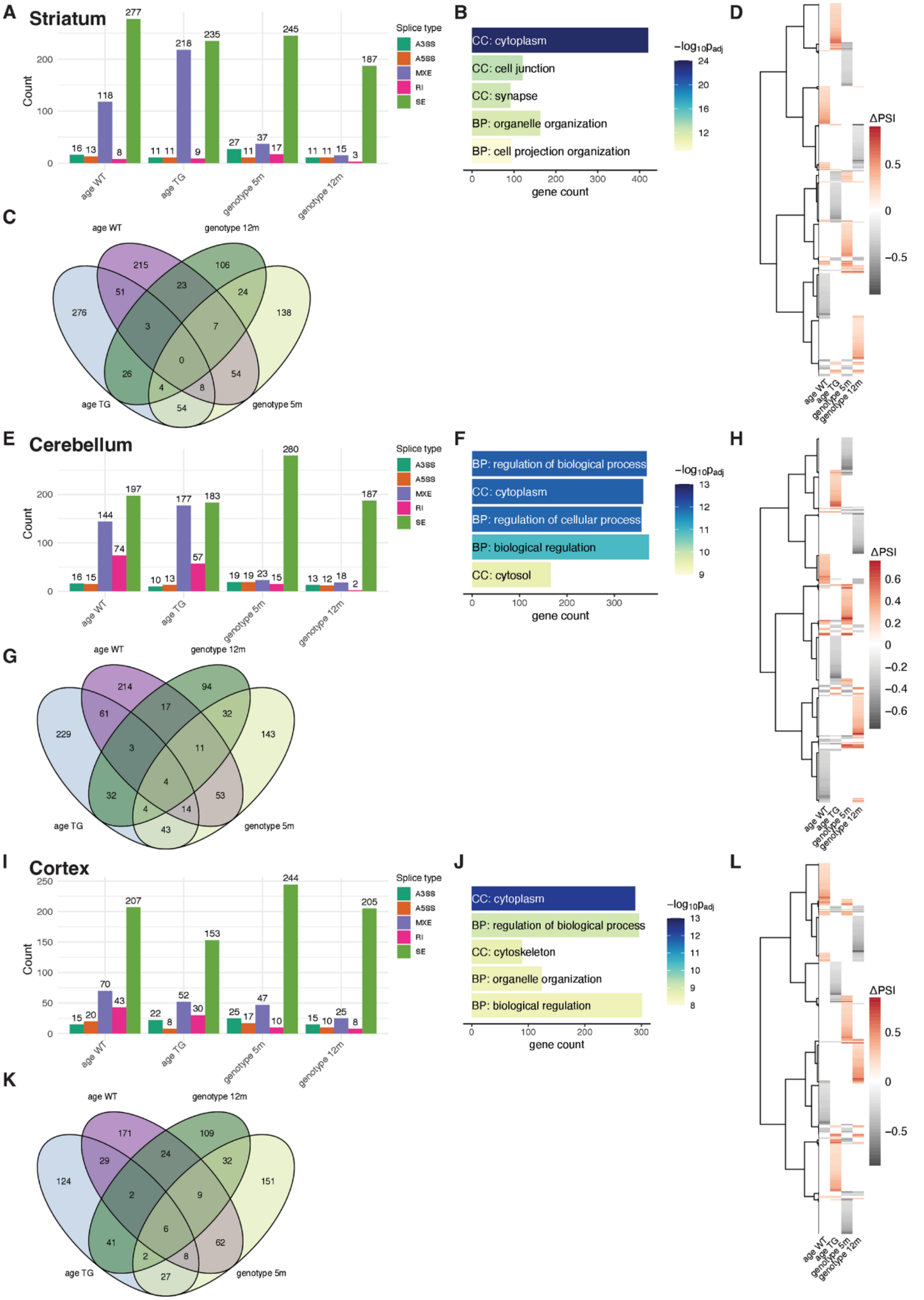

Supplementary Figure 18. Differential splicing events across brain regions and contrasts. (**A**, **E**, **I**) Differential splice event counts with respect to genotype and age. (age WT = WT_{12m}/WT_{5m}, age TG = TG_{12m}/TG_{5m}, genotype 5m = TG_{5m}/WT_{5m}, genotype 12m = TG_{12m}/WT_{12m}) for striatum (**A**), cerebellum (**E**), and cortex (**I**). Color code represents splice event type based on rMATs. Filtering of splice events based on ΔPSI (Percent Spliced In) ≥ 0.1 and FDR ≤ 0.01. (**B**, **F**, **J**) Five most significantly enriched Gene Ontology terms for differentially spliced genes from A, E, I for striatum (**B**), cerebellum (**F**), and cortex (**J**) with indicated adjusted p-value and gene count. (**C**, **G**, **K**) Venn diagram of genes with differential splicing events for striatum (**C**), cerebellum (**G**), and cortex (**K**). (**D**, **H**, **L**) Clustered heatmap of differential splicing events. Color-code reflects ΔPSI values. Heatmap limited to union of top 100 most significant (gene-)events per contrast.

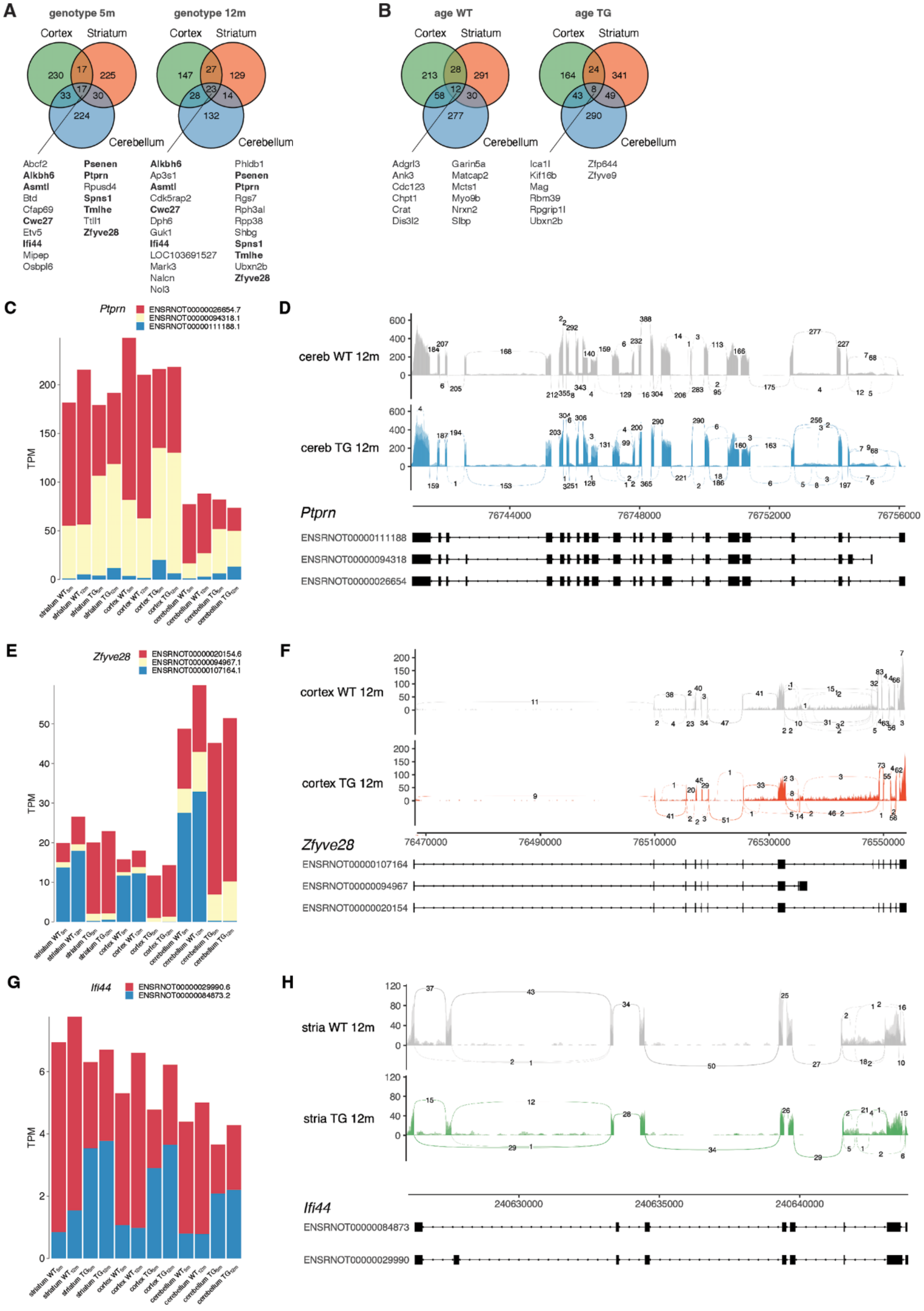

Supplementary Figure 19. Cross-regional differential splicing events with respect to age and genotype. (**A**) Venn diagram of differential splicing events across brain regions for WT and TG animals with respect to genotype. (**B**) Venn diagram of differential splicing events across brain regions for WT and TG animals with respect to age. (**C**, **D**) Isoform and Sashimi plot for *Ptprn* showing altered A3SS event (alternative 3' splice site) in TG mice. (**E**, **F**) Isoform and Sashimi plot for *Zfyve28* showing a MXE (mutually exclusive exon) towards the 3' end in transcript ENSRNOT00000107164.1 isoform and compositional shift in transcript isoforms between WT and TG animals. (**G**, **H**) Isoform and Sashimi plot for *Ifi44* showing a SE (skipped exon) event (2nd exon toward 5' end) and isoform compositional shifts between genotypes.

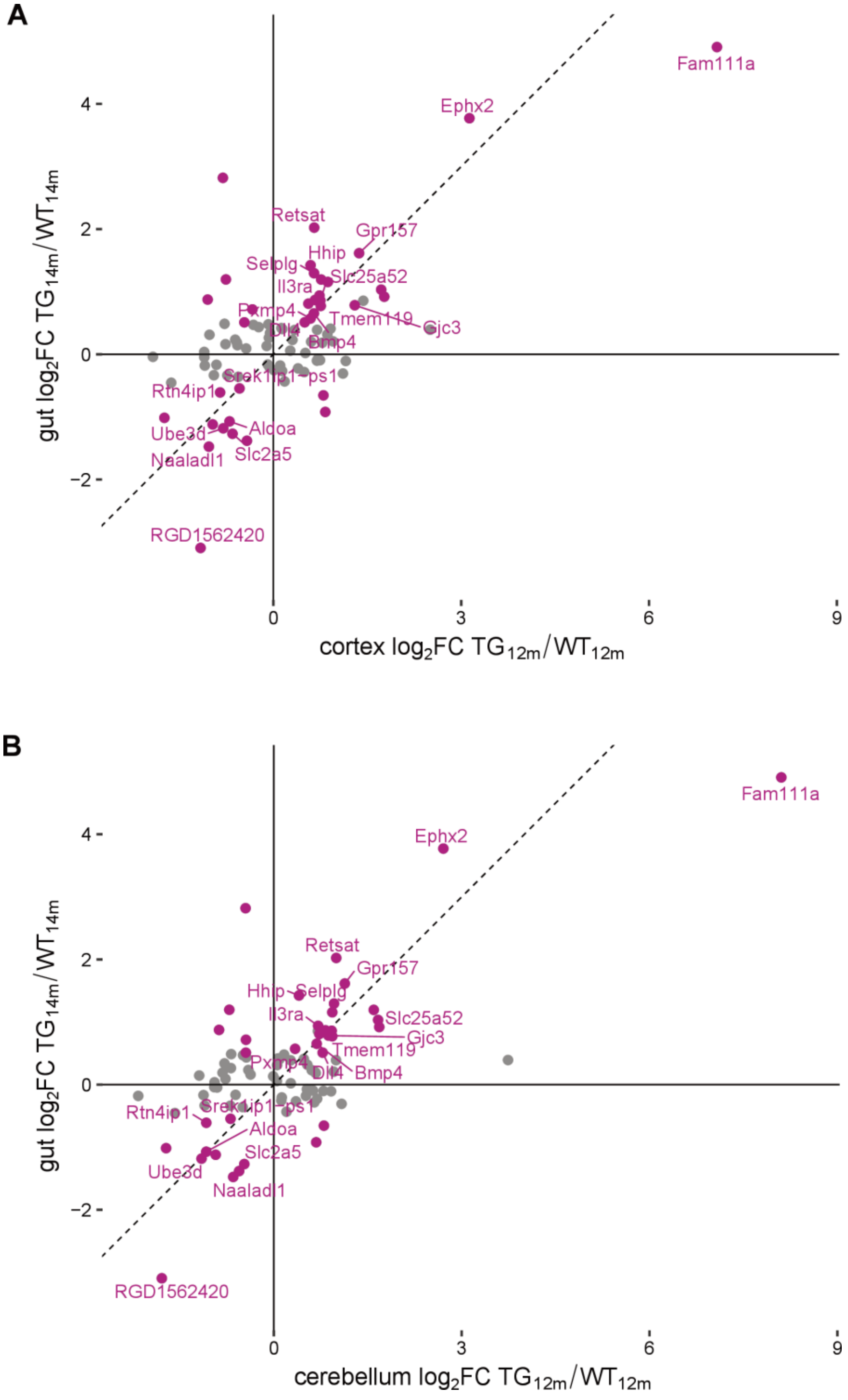

Supplementary Figure 20. Similar expression of core gene set in brain and gut. Scatter plot of 122 cross-regional DEGs in TG animals in cortex (**A**) or cerebellum (**B**) and gut, with 37 overlapping DEGs between brain regions and gut highlighted in purple. DEGs with the same regulation in brain and gut are labelled.
