# Supplementary material for: Brain region-specific and systemic transcriptomic alterations in a human alpha-synuclein overexpressing rat model
Source: Aging (Albany NY). 2025 Oct 20;17(10):2598–636. doi: 10.18632/aging.206331 (PMC12606970; doi:10.18632/aging.206331)
Supplement: Supplementary Table 2 [file aging-17-10-206331-s003.pdf]

Supplementary Table 2. Overlap of differential genes in prefrontal cortex of BAC SNCA rats at 12 months of age and differential proteins in human prefrontal cortex of PD patients.

rat ensembl gene ID	human ensembl gene ID	rgd symbol	hgnc symbol	protein accession	log2FoldChange_ protein_PD	padj_protein_PD	log2FoldChange _rat_WT-TG_12m	padj_rat_WT-TG_12m
ENSRNOG00000001404	ENSG00000106351	Agfg2	AGFG2	O95081	0.12219835	0.01660219	-0.535094	3.2558E-17
ENSRNOG00000059510	ENSG00000078053	Amph	AMPH	P49418	0.04792062	0.02058255	0.64313535	5.0338E-35
ENSRNOG00000010263	ENSG00000013297	Cldn11	CLDN11	O75508	-0.3066129	0.0341209	0.57397668	0.00000000000048484
ENSRNOG00000009563	ENSG00000172867	Krt2	KRT2	P35908	0.36652959	0.03009904	-1.1386355	0.00003809
ENSRNOG00000016346	ENSG00000163932	Prkcd	PRKCD	Q05655	-0.145365	0.00604063	-0.5225256	0.00000043932
ENSRNOG00000014090	ENSG00000042445	Retsat	RETSAT	Q6NUM9	-0.1450632	0.01291154	0.652551	0.00000039173
ENSRNOG00000058006	ENSG00000173267	Sncg	SNCG	O76070	0.10770146	0.01023248	2.50378994	9.4586E-64
